# A microsatellite DNA-derived oligodeoxynucleotide attenuates lipopolysaccharide-induced acute lung injury in mice by inhibiting the HMGB1-TLR4-NF-κB signaling pathway

**DOI:** 10.3389/fmicb.2022.964112

**Published:** 2022-08-04

**Authors:** Chenghua Zhang, Hui Wang, Hongrui Wang, Shuyou Shi, Peiyan Zhao, Yingying Su, Hua Wang, Ming Yang, Mingli Fang

**Affiliations:** ^1^Department of Molecular Biology, College of Basic Medical Sciences, Jilin University, Changchun, China; ^2^Department of Endoscopy, Jilin Provincial Cancer Hospital, Changchun, China; ^3^Department of Anatomy, College of Basic Medical Sciences, Jilin University, Changchun, China

**Keywords:** inflammation, NF-κB – nuclear factor kappa B, acute lung injury (ALI), high-mobility group box 1 (HMGB1), oligodeoxynucleotide (ODN), pathogen-associated molecular patterns (PAMPs)

## Abstract

Acute lung injury (ALI) with uncontrolled inflammatory response has high morbidity and mortality rates in critically ill patients. Pathogen-associated molecular patterns (PAMPs) are involved in the development of uncontrolled inflammatory response injury and associated lethality. In this study, we investigated the inhibit effect of MS19, a microsatellite DNA-derived oligodeoxynucleotide (ODN) with AAAG repeats, on the inflammatory response induced by various PAMPs *in vitro* and *in vivo*. In parallel, a microsatellite DNA with AAAC repeats, named as MS19-C, was used as controls. We found that MS19 extensively inhibited the expression of inflammatory cytokines interleukin (IL)-6 and tumor necrosis factor (TNF)-α induced by various PAMPs stimulation, including DNA viruses, RNA viruses, bacterial components lipopolysaccharide (LPS), and curdlan, as well as the dsDNA and dsRNA mimics, in primed bone marrow-derived macrophage (BMDM). Other than various PAMPs, MS19 also demonstrated obvious effects on blocking the high mobility group box1 (HMGB1), a representative damage-associated-molecular pattern (DAMP), nuclear translocation and secretion. With the base substitution from G to C, MS19-C has been proved that it has lost the inhibitory effect. The inhibition is associated with nuclear factor kappa B (NF-κB) signaling but not the mitogen-activated protein kinase (MAPK) transduction. Moreover, MS19 capable of inhibiting the IL-6 and TNF-α production and blocking the HMGB1 nuclear translocation and secretion in LPS-stimulated cells was used to treat mice ALI induced by LPS *in vivo*. In the ALI mice model, MS19 significantly inhibited the weight loss and displayed the dramatic effect on lessening the ALI by reducing consolidation, hemorrhage, intra-alveolar edema in lungs of the mice. Meanwhile, MS19 could increase the survival rate of ALI by downregulating the inflammation cytokines HMGB1, TNF-a, and IL-6 production in the bronchoalveolar lavage fluid (BALF). The data suggest that MS19 might display its therapeutic role on ALI by inhibiting the HMGB1-TLR4-NF-κB signaling pathway.

## Introduction

The innate immune system plays an important role in the control of pathogens, and it can be activated to produce a proinflammatory response by the recognition of pathogen-associated molecular patterns (PAMPs) *via* pattern recognition receptors (PRRs) (Li and Chang, 2021). The best-known examples of PAMPs include lipopolysaccharide (LPS) of Gram-negative bacteria and nucleic acids of pathogens. Among all the PRRs, Toll-like receptors (TLRs) have been studied most extensively. For example, it is known that TLR4 senses the LPS from bacteria, TLR2 recognizes polysaccharide and lipoproteins, TLR3 and TLR7/8 recognize viral double-stranded RNA (dsRNA) and single-stranded RNA (ssRNA), and TLR9 responds to microbial DNA rich in non-methylated CG motifs (CpGs), respectively (Mu and Hur, 2021). Upon activation, TLRs recruit a cytoplasmic protein, myeloid differentiation primary response protein 88 (MyD88), and subsequently bind the interleukin 1R-associated kinase (IRAK) complex. In turn, IRAK dissociates from the receptor–adapter complex upon phosphorylation and interacts with TNFR-associated factor 6 (TRAF6). Then, TRAF6 activates transforming growth factor activated kinase-1 (TAK1) and subsequently leads to the activation of two major pathways involving the Rel family transcription factor NF-κB and the mitogen-activated protein kinase (MAPK) family. The MAPKs mainly include the extracellular signal-regulated kinase (ERK) and p38. Activation of the MAPKs and NF-kB signaling pathways promotes inflammation by inducing the expression of interleukin (IL)-6, tumor necrosis factor-α (TNF-α), IL-1β, and some inflammatory chemokines (Park et al., 2022; Yue et al., 2022). In addition, interferon regulatory factor 5 (IRF5) is another downstream transcription factor in TLRs/MyD88 signaling pathways. TLRs activation triggers the formation of MyD88-IRF5-TRAF6 complexes and then activates IRF5, which leads to the IRF5 nuclear translocation to initial the transcription of proinflammatory cytokines (Tang et al., 2021).

Although inflammation can defend against invading pathogens, it is also a double-edged sword. Usually, deregulated or severe inflammation induced by PAMPs has been linked to tissue injury and inflammatory diseases, such as acute lung inflammatory injury, septic peritonitis, and multiple sclerosis (Zheng et al., 2019; Sun et al., 2022). Moreover, some endogenous molecules called damage-associated molecular patterns (DAMPs) could be passively released from the damaged cells of tissue injury (Zhou et al., 2021). High-mobility group box 1 (HMGB1) is a nuclear DNA-binding protein and acts as a typical DAMP molecule, which plays an important role in sterile inflammatory responses. HMGB1 can also be actively released from the nucleus to the extracellular space in response to different stimuli. The active secretion always requires the translocation of nuclear HMGB1 to the cytoplasm and the secretion of cytoplasmic HMGB1 to reach the extracellular space (Ding et al., 2021). Inside the nucleus, HMGB1 has been found to increase the binding affinity of many transcription factors to their cognate DNA sequences, such as NF−κB (Xue et al., 2021). More importantly, HMGB1 is also involved in extracellular activity as an endogenous danger signal, which participates in cell–cell interactions including the production of proinflammatory cytokines. For example, HMGB1 protein can interact with multiple cell surface receptors, including TLR2, TLR4, and TLR9, and subsequently activate NF−κB and IFN regulatory factor pathways, stimulating more production of cytokines and chemokines, aggravating the inflammatory response and tissue damage (Ge et al., 2021). Therefore, there is a need to precisely control and tightly regulate the TLRs-mediated signaling pathways induced by PAMPs and DAMPs for preventing the inflammation-induced pathology. Medicines that inhibit the activation of the TLRs signaling pathway are potential anti-inflammatory agents and may prevent tissue inflammatory damage.

In contrast to microbial DNA, host-derived DNA contents are usually considered as anti-inflammatory agents that could negatively regulate pathologic inflammatory responses (Hu et al., 2009; Bode et al., 2014; Herzner et al., 2016). In our previous study, an oligodeoxynucleotide (ODN) named as MS19, designed based on the sequence of human microsatellite DNA (MS DNA), with six AAAG repeated units. It could not only rescue mice from bacterial septic peritonitis (Gao et al., 2017) but also attenuate the acute lung injury (ALI) and myocarditis in virus-infected mice (Fang et al., 2011; Nie et al., 2019). It was confirmed that MS19 could reduce the expression of IRF5 and inhibit the nuclear translocation of IRF5 in RAW264.7 cells, since the sequence of AAAG unit is in consensus with the DNA-binding site of IRF5 (Gao et al., 2017). Moreover, MS19 also decreased the expression of IRF5 in the burn injury skin and the myocardial tissues of coxsackievirus B3-infected mice (Xiao et al., 2017; Nie et al., 2019). However, there are still more inflammatory signaling pathways that are involved in the regulation of TLR-induced inflammation, such as the NF-κB and MAPK signaling. Thus, it is necessary to explore the other potential anti-inflammatory mechanisms of MS19 except for the IRF5.

In this study, we assessed the inflammation inhibitory effect of MS19 against different pathogens and their component mimics, in parallel, an ODN with special base substitution, named as MS19-C, was used as a control. Moreover, the role of MS19 in the NF-κB and MAPK signaling, and the nucleocytoplasmic translocation and secretion of HMGB1 were evaluated in LPS-stimulated macrophages. Furthermore, a mouse model with LPS-induced ALI was established and used for observing the interfering effect of MS19 on inflammatory injury *in vivo*. The data obtained suggest that MS19 could drive the suppression of the NF-κB but not the MAPK signaling in PAMPs-induced inflammation, which provides a new insight into the mechanisms on how MS19 reduces the excessive inflammatory responses.

## Materials and methods

### Oligodeoxynucleotides

All oligodeoxynucleotides (ODNs) with nuclease-resistant phosphorothioate-modified, including the MS19 (5′-AAAGAAAGAAAGAAAGAAAGAAAG-3′) and the control ODN MS19-C (5′-AAACAAACAAACAAACAAACAAAC-3′), were synthesized in the Takara Biotechnology Company (Dalian, China).

### Virus, cell isolation, and culture

A mouse-adapted H1N1 influenza virus PR8 and a plaque-purified herpes simplex virus-1 (HSV-1) strain were obtained from the Department of Microbiology, Jilin University, Changchun, China. Virus stocks were propagated in Vero cells, which were then aliquoted and stored at −80°C. All experiments involving pathogens were handled at the laboratory with biosafety level 2 (BSL-2).

Mouse bone marrow-derived macrophages (BMDMs) were isolated as previously described (Bailey et al., 2020). Briefly, mice legs were cut to access the femur and tibia, and muscle and tissue were removed in sterile environment. The bone marrow content was washed out using a syringe with complete RPMI 1640 (GIBCO, Shanghai, China). The cells were centrifuged at 1,000 rpm for 5 min, and the pellet was resuspended in complete RPMI 1640 with 20% L929 culture supernatant. The media was replaced every 3 days. The experiment was performed on the 7th day of differentiation. The RAW264.7 cell line was obtained from the Cell Bank of Chinese Academy of Sciences (Shanghai, China). Cells were cultured at 37°C in a 5% CO_2_ humidified incubator and maintained in RPMI 1640 medium supplemented with 10% fetal bovine serum (HyClone, Logan, UT, United States) and antibiotics (100 IU/ml penicillin and streptomycin).

### Pathogen-associated molecular patterns stimulation and oligodeoxynucleotide transfection in cells

Bone marrow-derived macrophage and RAW264.7 cells were planted into 6-well plates at a density of 3 × 10^5^ cells per well and then treated with 1 μg/ml LPS (Sigma-Aldrich, St. Louis, MO, United States), 5 μg/ml VACV-70 (InvivoGen, Hong Kong), 25 μg/ml long polyI:C (LPIC), 20 μg/ml curdlan, or infected with PR8 or HSV-1virus at MOI 10:1 in the presence or absence of 2 μg/ml ODNs, respectively. Transfection of ODNs was performed using Lipofectamine 3000 (Thermo Fisher Scientific, Carlsbad, CA, United States) according to the manufacturer’s instructions. After 30 min of treatment, the cells were harvested to analyze the total expression and phosphorylation analysis of proteins in the cells, which were detected using Western blot. After 16 h of treatment, the cell supernatants were harvested for TNF-α, IL-6 and HMGB1 expression analysis by enzyme-linked immunosorbent assay (ELISA) kits (R&D Systems, Minneapolis, MN, United States) according to the manufacturer’s instructions.

### Western blot analysis

Protein extraction solution (Beyotime Biotechnology, Shanghai, China) was added to the cultured cells. The protein concentration was determined using a BCA Protein Assay Kit (Solarbio). Equivalent amounts of protein (20 μg) were loaded onto a gel and separated by sodium dodecyl-sulfate polyacrylamide gel electrophoresis (SDS-PAGE) and then transferred to nitrocellulose membranes (Thermo fisher scientific). The membranes were blocked in 5% bovine serum albumin (BSA) in Tris-buffered saline/Tween 20 at room temperature for 1 h, followed by incubation overnight at 4°C with rabbit polyclonal antibodies against p-p65, p65, p-p38, p38, p-ERK, ERK, p-TAK1, TAK1, and β-actin, all antibodies were obtained from the Cell Signaling (Beverly, MA, United States). After washing, the membrane was incubated with horseradish peroxidase-conjugated goat anti-rabbit IgG (Beyotime; 1:3,000 dilution). Immunoreactivity was visualized by enhanced chemiluminescence (Thermo fisher scientific), and signals were quantified using the ImageJ software (National Institutes of Health, Bethesda, MD, United States) or film autoradiography.

### Immunofluorescence

RAW264.7 cells were seeded in 4-well chambered glass slides coated with poly-L-lysine (Sigma-Aldrich). Treatment dosage for LPS and/or MS19 was adjusted to facilitate visualization *via* immunofluorescence. After washing with ice-cold PBS (pH 7.4), the cells were fixed using 4% paraformaldehyde for 30 min. Subsequently, the cells were further washed three times in PBS containing 0.1% Triton X-100, followed by incubation with 3% BSA in PBS for 30 min. Thereafter, the cells were incubated with primary rabbit antibodies against HMGB1 (1:200; Abcam, Shanghai, China) at 4°C overnight. After washing with PBS, cells were incubated with goat anti-rabbit IgG against HMGB1 conjugated with FITC (1:200; Beyotime) for 1 h at room temperature. Finally, the nuclei were counterstained with 4,6-diamidino-2-phenylindole (DAPI), and the coverslips were placed. Immunostaining was analyzed using a fluorescence microscope (Nikon) interfaced with a digital charge-coupled device camera and an image analysis system. The nucleocytoplasmic translocation percentage in each group was calculated under three fields of vision in fluorescence microscope.

### Lipopolysaccharide-induced acute lung injury mouse model

Eight-week-old specific pathogen-free female BALB/c mice (18 ± 2 g) were obtained from the Vital River Laboratory Animal Technology Co., Ltd. (Beijing, China). The mice were maintained at 22 ± 2°C with a 12 h light/dark cycle and had free access to food and water for experiments in accordance with the National Institute of Health Guide for the Care and Use of Laboratory Animals. All mouse experiments were approved by the ethics committee of the College of Basic Medical Sciences of Jilin University. Briefly, mice were randomly assigned to three study groups, namely, PBS, LPS, and LPS plus MS19, with 11 mice in each group. To study the inhibitory effect of MS19 on the LPS-induced lung inflammation, LPS was administered *via* intranasal injection. Mice were intraperitoneally anesthetized with pentobarbital sodium (50 mg/kg) and then were injected *via* the tail vein with PBS or MS19 (25 μg/mouse) at 6 h before LPS (20 mg/kg) inoculation. It was worth noting that MS19 was encapsulated in N-[1-(2,3-dioleoyloxy) propyl]-N, N, N-trimethylammonium methyl-sulfate (DOTAP) (Roche) in animal experiments. Part of the mice (three mice per group) were euthanized using CO_2_ at 1 day after exposure to LPS, and then the bronchoalveolar lavage fluid (BALF) samples were collected; after centrifuging at 3,000 rpm for 10 min at 4°C, the supernatant of the BALF samples were used for the ELISA analysis. Subsequently, the lungs were isolated for histopathological examination. Finally, the survival and weight of the rest mice (six mice per group) were recorded daily for 15 days after LPS injection.

### Lung histological assessments

The lung tissues with 4% paraformaldehyde were embedded in paraffin. Paraffin sections, 8 μm thick, were deparaffinized, rehydrated, stained with hematoxylin and eosin (H&E), and viewed under a microscope. A pathologist who was blinded to the data performed the histological assessments based on a method described previously (Jiang et al., 2021). The assessment was scored on a scale of 1 (i.e., normal) to 5 (i.e., maximal). The degree of leukocyte infiltration in peribronchiolar areas was judged by the number of infiltrated leukocytes. The sum of the score of cell infiltration and damage levels, including the thickening of the alveolar walls and epithelium, yielded the lung inflammatory score.

### Statistical analysis

Statistical analyses were performed using the SPSS v.19.0 software (IBM, Armonk, NY, United States). Quantitative data are presented as the means ± SEMs. All data were tested for normality and homoscedasticity. Differences between groups were evaluated using the Student’s *t*-test and one-way analysis of variance (ANOVA) followed by the Tukey–Kramer’s multiple range test. Survival rates of mice were compared using the Kaplan–Meier test. *P* < 0.05 was considered significant.

## Results

### MS19 inhibited the expression of inflammatory cytokines induced by various pathogen-associated molecular patterns

**FIGURE 1:**
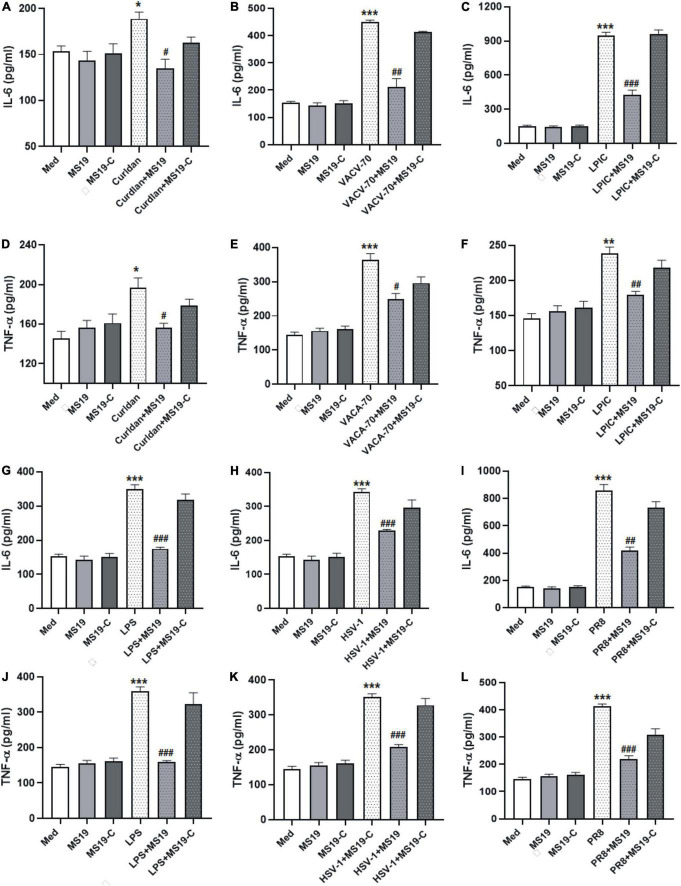
MS19 plays an inhibitory role on regulating interleukin 6 (IL-6) and tumor necrosis factor-α (TNF-α) production in bone marrow-derived macrophage (BMDM) cells upon various pathogen-associated molecular patterns (PAMPs) stimulation. BMDMs were treated with different stimulators in the presence or absence of MS19, and then culture supernatants were collected to evaluate the expression of IL-6 and TNF-α level using ELISA. The cytokine levels of IL-6 in BMDM supernatants with the stimulation of bacterium components curdlan, dsDNA virus mimic VACV-70, and RNA virus mimic LPIC **(A–F)**. The cytokine levels of IL-6 and TNF-α in BMDM supernatants with the stimulation of LPS **(G,J)**, DNA virus HSV-1 **(H,K)**, and RNA virus PR8 **(I,L)**. The values are presented as the means ± SEM of three independent experiments. *p < 0.05, **p < 0.01, and ***p < 0.001, vs. medium group; ^#^*p* < 0.05, ^##^*p* < 0.01, and ^###^*p* < 0.001, vs. curdlan, VACV-70, LPIC, LPS, HSV-1, or PR8.

Pathogen-associated molecular patterns (PAMPs) that are present during infection can induce lung inflammatory responses and injuries. To evaluate the inhibitory effect of MS19 on different PAMPs-induced inflammation, the production of IL-6 and TNF-α in BMDM stimulated with various different PAMPs stimulants were analyzed using ELISA. We chose the bacterial components (i.e., LPS and Gram-positive bacteria cell wall component Curdlan), DNA viruses Herpes simplex virus (HSV) and DNA virus mimics (VACV-70), RNA virus influenza virus (PR8), and RNA virus metabolism products dsRNA long poly (I: C) (LPIC) as stimulus. MS19 (1 μM) or MS19-C (1 μM) transfected with Lipofectamine 3000 (Lipo3000) into cells and then incubated with different stimulus for 16 h. The concentration of TNF-α and IL-6 in the supernatant was detected by ELISA. The results showed that MS19 could significantly inhibit IL-6 and TNF-α production from BMDM triggered Curdlan, DNA virus mimics VACV-70, and RNA virus mimics LPIC (Figures 1A–F). Similarly, the suppressive effects of MS19 were also observed on the expression of inflammatory cytokines IL-6 and TNF-α induced by bacterial LPS, DNA virus, and RNA virus in BMDM (Figures 1G–L). Those results showed that MS19 could significantly inhibit the expression of inflammatory cytokines induced by various PAMPs in macrophages. Interestingly, with the base substitution from G to C, MS19-C has been proved that it has lost the inhibitory effect against the different PAMPs-induced inflammation, which indicated that the inhibitory effect of MS19 was in a sequence-specific manner.

### MS19 drove the suppression of NF-κB but not the mitogen-activated protein kinase signaling in pathogen-associated molecular patterns-induced inflammation

**FIGURE 2:**
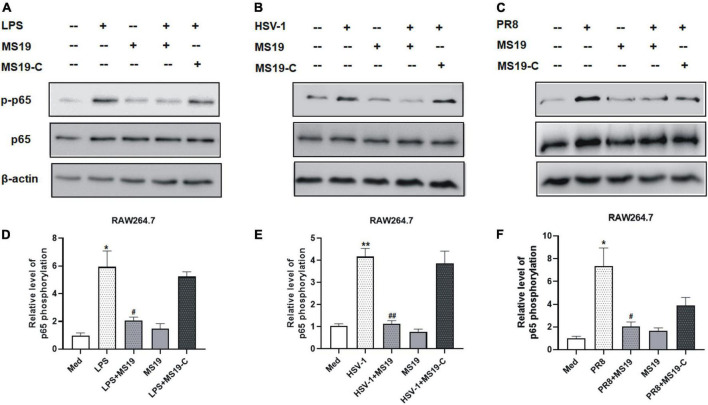
Inhibitory effect of MS19 on the expression of NF-κB signaling pathway in different PAMPs-stimulated RAW264.7 cells. The protein expression of p-P65, p65, and β-actin in RAW264.7 cells stimulated by LPS, HSV-1, and PR8 were detected by Western blot **(A–C)**. Band intensity was measured using an imaging densitometer, and the expression of the proteins was calculated relative to the intensity of β-actin protein **(D–F)**. Western blot analysis was assayed in triplicate for each sample. Data represent the mean ± SEM. *p < 0.05, **p < 0.01, vs. medium group; ^#^*p* < 0.05, ^##^*p* < 0.01, vs. LPS, HSV-1, or PR8 group.

Based on the inhibition features of MS19 against various kinds of PAMPs, we deduced that MS19 should inhibit some common molecular pathways among all the inflammatory signaling. To further investigate the possible mechanisms of MS19 to inhibit the PAMPs-induced inflammation, we focused on the NF-κB and MAPK signaling pathways of the TLR4 downstream pathway, which are the most extensively investigated inflammatory signaling pathways. Therefore, we first examined the effects of MS19 on the phosphorylation levels of p65 NF-κB in the presence of different stimuli including LPS, HSV-1, and PR8 in RAW264.7 cells. As reflected in Figure 2, stimulation with these PAMPs increased the phosphorylation level of p65 in RAW264.7 cells, whereas co-treatment with MS19 significantly reduced the upregulation.

**FIGURE 3:**
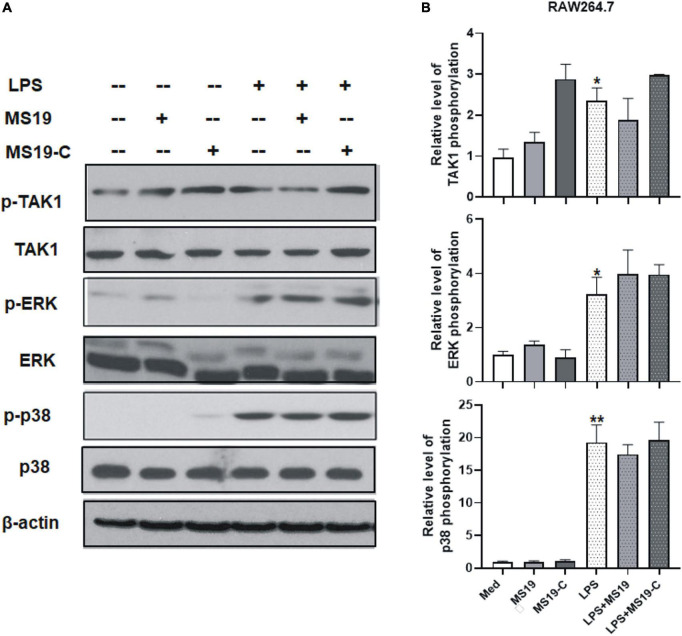
Effect of MS19 on the expression of MAPK signaling pathway in LPS-stimulated RAW264.7 cells. The protein expressions of p-TAK1, TAK, p-ERK, ERK, p-p38, p38, and β-actin in RAW264.7 cells were detected by Western blot **(A)**. Band intensity was measured for calculating the expression of the proteins relative to the intensity of β-actin protein **(B)**. Data represent the mean ± SEM. *p < 0.05, **p < 0.01, vs. medium group.

To determine if LPS/TLR4 downstream MAPK signaling pathways were activated, we measured the levels of nuclear phosphorylated mitogen-activated protein (MAP) kinases in LPS-treated peritoneal macrophages by Western blot. The results showed that the phosphorylation of TAK-1, ERK, and p38, the activation of major constitutes of MAPKs family, had no obvious changes with MS19 treatment in LPS-stimulated cells (Figure 3). Comparatively, the total protein expression of TAK-1, ERK, and p38 in cells were not affected by the stimulation and treatments. These results indicated that MS19 exhibited potential anti-inflammatory effects *via* inhibiting the NF-κB but not the MAPK signaling pathways in macrophages.

### MS19 suppressed the nucleocytoplasmic translocation and secretion of HMGB1 in lipopolysaccharide-stimulated macrophages

**FIGURE 4:**
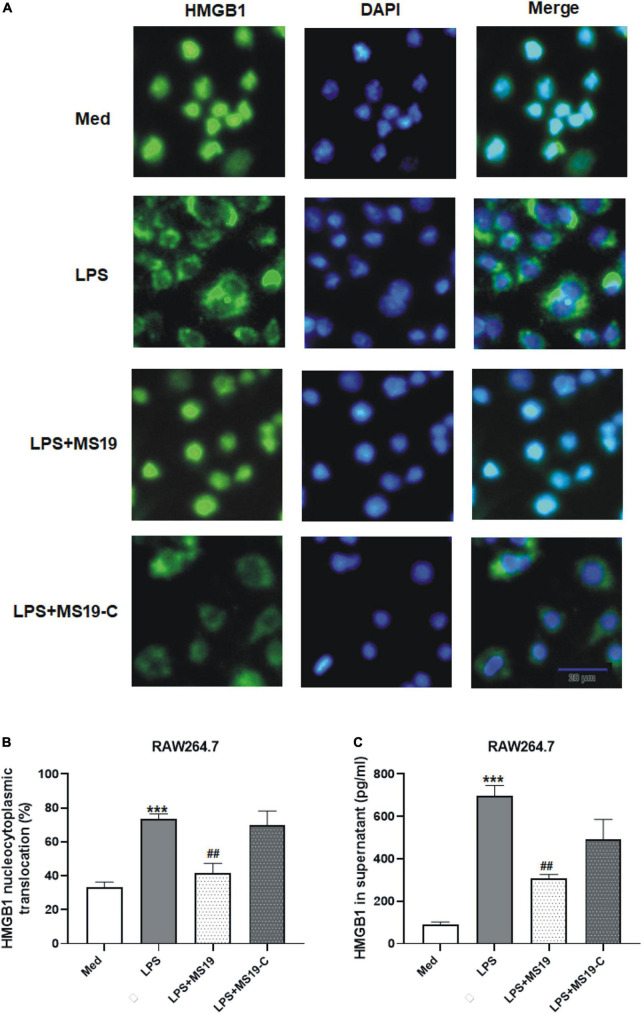
Suppression of HMGB1 translocation and secretion in RAW264.7 cells treated with MS19 after LPS exposure. Immunofluorescence images of nucleocytoplasmic translocation of HMGB1 **(A)**. RAW264.7 cells were incubated with LPS with or without MS19 for 2 h and examined by fluorescence microscopy. HMGB1 are stained in green with FITC, and cell nuclei are stained in blue with DAPI. The percentage of nucleocytoplasmic translocation of HMGB1 in RAW264.7 cells was calculated **(B)**. The HMGB1 level in supernatant after RAW264.7 cells received 16 h exposure of LPS in the presence or absence of MS19, as determined using ELISA **(C)**. Data are representatives of three independent experiments and are expressed as means ± SEM. ***p < 0.001, vs. medium group; ^##^*p* < 0.01, vs. LPS group.

HMGB1 is important in the initiation and progression of proinflammatory processes. It could be released into the extracellular environment during exogenous stimulus, such as LPS. Some non-immunogenic nucleotides with high-affinity HMGB binding may function as suppressing agents for HMGB-mediated inflammation disease by blocking TLR4 activation and macrophage cytokine release (Yang et al., 2010; Yanai et al., 2011). Next, we evaluated the inhibitory effects of MS19 on the nucleocytoplasmic translocation and secretion of HMGB1 in LPS-stimulated RAW264.7 cells using immunofluorescence assay and ELISA. As shown in Figures 4A,B, HMGB1 was strictly located in nucleus of untreated macrophages, and the translocation of HMGB1 from the nucleus to cytoplasm was happened when the cells were exposed with LPS (*P* < 0.001). However, MS19 displayed a strong suppressive effect on HMGB1 translocation in LPS-stimulated cells (*P* < 0.01). Furthermore, LPS-induced high HMGB1 level in supernatant was also inhibited by treating with MS19 (*P* < 0.01) (Figure 4C). Similar to the expression of inflammatory cytokines, no obvious difference of HMGB1 nucleocytoplasmic translocation and secretion was observed in RAW264.7 cells between the group treated with LPS and LPS + MS19-C. The data demonstrated that MS19 inhibited the translocation of HMGB1 to the cytosol, thereby reducing its active secretion.

### MS19 alleviated the lipopolysaccharide-induced acute lung injury and mortality in mice

**FIGURE 5:**
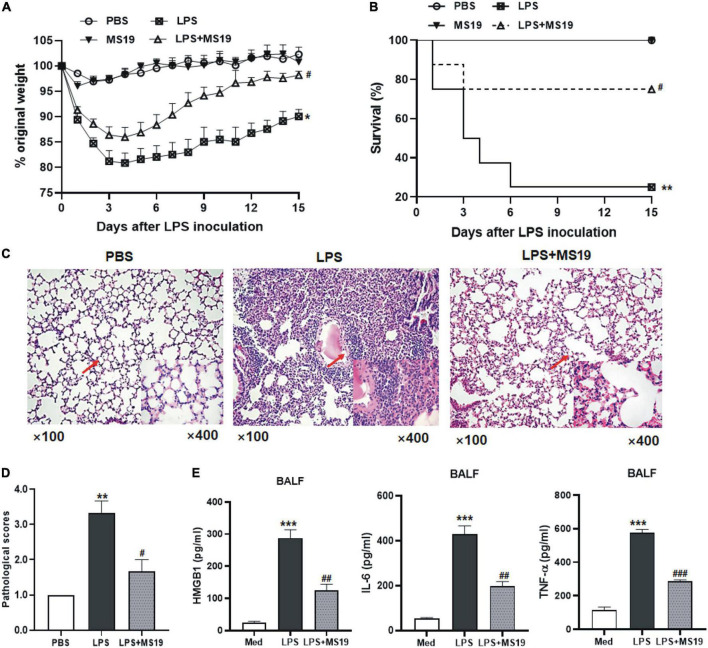
Protective effect of MS19 on LPS-induced acute lung injury in mice. Mice (*n* = 11) were challenged nasally with LPS or PBS at 6 h after intravenous injection with MS19 or PBS. The body weight change of mice **(A)**. The percent survival of mice in different groups at 8 days after LPS challenge **(B)**. Representative images of lung pathology after H&E staining **(C)**. Lung injury scores **(D)**. ELISA of HMGB1, IL-6, and TNF-α level in the BALF samples of mice in each group **(E)**. Data represent the mean ± SEM. *p < 0.05, **p < 0.01, and ***p < 0.001, vs. PBS group; ^#^*p* < 0.05, ^##^*p* < 0.01, and ^###^*p* < 0.001, vs. LPS group.

Next, we evaluated the inhibitory effect of MS19 on the acute lung pathological inflammation caused by LPS in mice. These mice were treated with LPS or/and MS19 on day 1, then three mice of each group were euthanized, and BALF was collected from the lungs, and then, the lungs were removed for histopathological examination at day 2. The survival and weight of the rest eight mice were recorded daily for 8 days. As shown in Figure 5A, LPS administration could induce a dramatic loss of body weight in mice, nearly 20% weight loss in the LPS group at day 4. The weight of the mice in MS19 alone group was no change until the end of experiment. However, in the LPS + MS19 group, LPS stimulation induced an initial dramatic decrease 1–3 days after injection, followed by recovery at day 4 and a further increase at day 5. From day 8 post injection, the body weight of the mice treated with LPS + MS19 was gradually recovered toward the normal until at the end of experiment at day 15, whereas the body weight of the mice treated with LPS was not recovered resulted in only two mice were lived at day 8. Six of the eight mice in the LPS + MS19 group were still alive (*P* < 0.05) (Figure 5B). The mortality rates of the mice treated with PBS or MS19 alone or LPS alone or LPS + MS19 were 100, 100, 25, or 75%, respectively, indicating the more potential of MS19 on inhibiting lung inflammation responses.

Lung pathological results showed that the mice stimulated with LPS showed thickened and congested alveolar walls, intra-alveolar edema, and numerous infiltrated neutrophils and macrophages in their lung tissues. In contrast, mice treated with MS19 showed no or much less pathological changes in their lung tissues (Figure 5C). The pathological score of the lung tissues in MS19-treated mice was lower than that in mice inoculated with LPS only (*P* < 0.05) (Figure 5D). In addition, the secretion of HMGB1, TNF-a, and IL-6 in the BALF of mice treated with LPS was significantly higher than that of the PBS mice, while the levels of these inflammatory mediator levels in the mice treated with MS19 were obviously lower than that in LPS group (Figure 5E). The results indicated that MS19 has a potential protection against the lethal LPS-induced ALI by inhibiting the production of inflammatory cytokines.

## Discussion

Excessive TLR signaling may lead to persistent inflammation and tissue destruction. For instance, severity of the coronavirus disease 2019 (COVID-19) is associated with cytokine storm in patients, which could be produced by over-activation of TLR pathways (Tang et al., 2020; Manik and Singh, 2021). Therefore, it is quite obvious that targeted manipulation of TLR signaling pathway may possess the decrease of excessive inflammatory responses. In this study, we found that MS19 could inhibit the various PAMPs-induced inflammatory cytokines remarkably, and extensively, and the inhibition is associated with NF-κB signaling pathway.

Multiple pathogens and their PAMPs were used to stimulate macrophages in this study. Various PAMPs trigger inflammatory responses may interact with different innate immune receptors. For example, pathogen lipoprotein and polysaccharide sensed by TLR2, bacterial LPS recognized by TLR4, viral and bacterial DNA with unmethylated CpG-DNA motifs sensed by TLR9, viral ssRNA and dsRNA could be recognized by TLR7/8 and TLR3, respectively (Naqvi et al., 2022). Thus, one virus particle can activate a complex pattern of TLRs, for instance, influenza A virus could be recognized by a bunch of TLRs including TLR2, -3, -4, and -7/8 (Pulendran and Maddur, 2015; Dai et al., 2018). Our result reflected that MS19 extensively inhibited the production of IL-6 and TNF-α induced by various PAMPs, which including the LPS, curdlan, dsRNA, and dsDNA mimics, as well as DNA and RNA virus. Moreover, in our previous published articles, we demonstrated that MS19 could inhibit the IFN-α production in human PBMC and pDC stimulated with RNA virus and DNA virus (Sun et al., 2010). Those results hinted toward not a direct interference at the receptors; therefore, we deduced that the MS19 might target one or more molecules of co-existing in the downstream of TLRs signaling. Three major signaling pathways, namely, NF-κB, MAPKs, and IRFs, are responsible for mediating TLRs-induced proinflammatory responses (Yang et al., 2010). In our previous study, MS19 was thought to bind with IRF5 because of the consensus AAAG repeat−binding site and prevented the nuclear translocation of IRF5 with subsequent induction of inflammatory genes (Xiao et al., 2017). However, considering the complexity of inflammation, it was possible that there still have additional targets for MS19 to act its anti-inflammation effect. Just like A151, an ODN containing 4 repeats of the TTAGGG motif, which was initially identified as a TLR9 antagonist that inhibits immune activation by CpG ODNs (Steinhagen et al., 2018). However, subsequent studies have found A151 also can bind to the cytosolic DNA sensors AIM2 and compete with DNA to inhibit cyclic GMP-AMP synthase (cGAS) pathway, resulting in the inflammasome inhibition (Eichholz et al., 2016; Steinhagen et al., 2018).

In this study, we focused on the TLR4 signaling pathways in macrophage induced by LPS to investigate whether MS19 could inhibit the NF-κB or MAPK transduction. Excitingly, MS19 significantly inhibited the phosphorylation levels of p65 NF-κB in LPS-stimulated cells. This finding was a supplement for the inflammatory inhibition mechanism of MS19. By means of mechanism, MS19 inhibited the TNF-α production in macrophage stimulated with LPS by blocking the HMGB1 translocation and secretion. LPS activation of TLR4 and caspase-11 both generate extracellular HMGB1 release. TLR4 activates both the MyD88-dependent and TRIF-dependent pathways (Lu et al., 2008). HMGB1-induced TNF-α production is TRIF and MyD88-dependent pathway, consistent with what is known regarding LPS downstream signaling (Kim et al., 2013). qRT-PCR results showed that MS19 reduced the overexpression of TRAF6, MyD88, and IRF3 mRNA caused by LPS in RAW264.7 (Supplementary Figure 1 and Supplementary Table 1). In our unpublished results, MS19 also could inhibit the IFN-α and IFN-β production in macrophage and pDC stimulated with DNA and RNA virus. TRIF-signaling downstream of LPS-bound TLR4 leads to the activation of the IFN-regulatory factors IRF3/7 (Fitzgerald et al., 2003) and produce the IFN-β and IFN-α. The type I interferon expression was TRIF dependent and did not require MyD88. So, we speculated that MS19 may inhibit the TNF-α release both in TRIF- and MyD88-dependent pathway. Besides TLR4, caspase-11 is also drive inflammatory response by mediated the HMGB1 translocation from the nucleus to the cytoplasm. LPS induced activation of caspase-11 in macrophages, leading to reduced ASC speck formation, caspase-1 activation, matured IL-1β release. But, MS19 did not affect the proinflammatory cytokines IL-1 level in macrophage stimulated with LPS (data not shown). We speculated that the inhibition of MS19 on HMGB1 translocation in macrophage may be dependent on TLR4, independent of caspase-11. About the TLR4 downstream signaling pathway, treatment of RAW264.7 cell with MS19 did not affect the phosphorylation and expression of TAK1, ERK, and p38 MARK. Thus, MARKs pathway was not involved in the inflammatory regulation of MS19. Surely, besides the TLRs signaling, there still have more pathways are involved the inflammation, such as the cytosolic sensor signaling including the nucleotide-binding and oligomerization domain-like receptors (NLRs), the retinoic acid-inducible gene 1-like receptors (RLRs) and the cGAS (Decout et al., 2021; Kienes et al., 2021; Pu et al., 2022). Thus, more experiments are still needed to understand the full inhibition mechanisms of MS19 in the future.

The effect of all inhibitory ODNs is associated with high sequence specificity. For instance, the sequences of antisense oligodeoxynucleotides are limited to pair with the specific target RNA (Alharbi et al., 2020). Moreover, among the traditional suppressive ODNs, INH-ODN 2088, and A151, the inhibitory effect against TLRs signaling was based on the motif of CC(T)XXX 3–5 GGG or TTAGGG, respectively (Zhang et al., 2015; Steinhagen et al., 2018). To investigate the key base of MS19 in the inhibitory effect, we substituted all the guanine (G) to cytosine (C) to form the control ODN MS19-C. The results showed that the MS19-C had no more inhibitory activities in triggering the expression of IL-6 and TNF-α, as well as the activation of NF-κB signaling after different PAMPs stimulation. These data indicated that the G-rich ODN may exhibit a stronger inhibition ability than non-G ODN. This finding was consistent with a previous study, which identified a G-rich ODN with ploy G motif (G-ODN), could block the secretion of TNF-a and IL-12p40 and interfere with the upregulation of major histocompatibility complex (MHC) class II and costimulatory molecules (Peter et al., 2008). Interestingly, certain G-rich DNA sequences can fold into a variety of four-stranded structures, which are called G-quadruplexes (Zuffo et al., 2018). The sequence feature of MS19 has six G bases in it and with a specific interval, which may facilitate to form the G quadruplex-like structures. Compared with the non-G quadruplex ODN, G quadruplex ODN blocked more efficiently TLR7- and TLR9-mediated innate immune responses (Römmler et al., 2013). This phenomenon might be related with the regulatory role of G quadruplex in inflammatory-related gene promoters (Stein and Eckert, 2021). However, more is not always better; some new evidence suggested that inhibitory ODNs containing many G triplets, or quadruples were easy to form higher order structures, also called G4 stacks, which made their immunological and pharmacological behaviors unpredictable (Lenert, 2010; Römmler et al., 2013; Zuffo et al., 2018). Therefore, keep the appropriate number of G base or with the G modification allows the development of inhibitory ODNs with superior inhibitory potency for inflammatory diseases (Römmler et al., 2013).

HMGB1 is an evolutionarily conserved protein, presenting in the nucleus of eukaryotic cells under basal conditions. Generally, the secretion of extracellular HMGB1 by innate immune cells in response to PAMPs or released by injured cells has been identified to play a key role in the pathogenesis of sterile and infectious inflammation (Ueno et al., 2021). The release of HMGB1 was confirmed in our study. LPS induced the nucleocytoplasmic translocation, and secretion of HMGB1 was decreased by treating with MS19. The HMGB1 release is mediated by the nuclear export protein XPO1 and secretory lysosomes, which can be triggered by a variety of cytokines and signaling pathways. Some reports have showed that the inhibition of the NF-κB signaling pathway limits HMGB1 secretion in activated immune cells (Li et al., 2020; Chen et al., 2022). Although the target of NF-κB signaling directly responsible for this process is still unclear, a possible mechanism of that is the TNF-α production. The reason is that the release TNF-α and HMGB1 in a time-dependent manner with response to LPS stimulation in macrophages and monocytes. Furthermore, the direct TNF-α suppression *via* gene knockout or TNF-α neutralizing antibodies partially inhibited LPS-induced HMGB1 release in macrophages (Chen et al., 2004; Chen et al., 2022). Therefore, we speculated that the inhibition role of MS19 on HMGB1 secretion is partially mediated through NF-κB signaling and TNF-α-dependent mechanism, since that ODN exhibited a strong inhibition against the NF-κB activation and TNF-α production in this study. In addition, after its release, extracellular HMGB1 usually acts as a DAMP molecule and transmits signals to the cell interior *via* the activation of receptors including TLR4, resulting in the formation of a positive feedback loop that potentially amplifies local inflammatory responses (Su et al., 2021; Teo Hansen Selno et al., 2021). Thus, MS19 might also interact with the secreted HMGB1, leading to the functional inactivation by the induction of a conformational change. Three critical elements of ODNs, namely, phosphorothioate modification, base sequence, and length of ODN, were identified for their high-affinity binding to HMGB1 (Yanai et al., 2011). The MS19 used in this study has phosphorothioate modification and potential G-quadruple structure, thereby resulting in the possibility of HMGB1 inactivation through direct binding.

In animal experiments, a classic experimental model of ALI induced by LPS has been used in this study. LPS results in symptoms and inflammatory response in animal models, closely resembling ALI in human (Ehrentraut et al., 2019). Following the LPS challenge, we observed that MS19 suppressed the LPS-induced HMGB1, TNF-α, and IL-6 inflammatory cytokines in the BALF of mice, which is consistent with the results in cultured macrophages. Moreover, MS19 exhibited the anti-inflammatory effects on the pathological changes of ALI, for example, the thickened alveolar walls and numerous infiltrated inflammatory cells were markedly reduced. The decrease of pathological changes is tightly associated with the inhibition of inflammatory cytokines and HMGB1 release (Lai et al., 2016). More importantly, MS19 significantly improved the survival rate of mice in LPS-induced ALI model, which is similar with the previous animal results of MS19 in rescuing the mice from bacterial septic peritonitis, burn-induced systemic inflammation, and coxsackievirus B3 (CVB3)-induced myocarditis (Gao et al., 2017; Xiao et al., 2017; Nie et al., 2019). Those data provided a supplemental experimental basis for the therapeutic applications in the treatment of numerous inflammatory diseases. It is noteworthy that MS19 inhibited the inflammatory response through the NF-κB signaling but not the MAPK transduction; this inhibition would be specific. Sometimes, a specific inhibition should be favorable, the MAPK signaling is known to be involved in many regulatory pathways (Mathien et al., 2021); thereby, the lack of suppression of MAPKs by MS19 might avoid the potential danger of side effects such as increasing infection rates as a result of excess immunologic suppression.

## Conclusion

**FIGURE 6:**
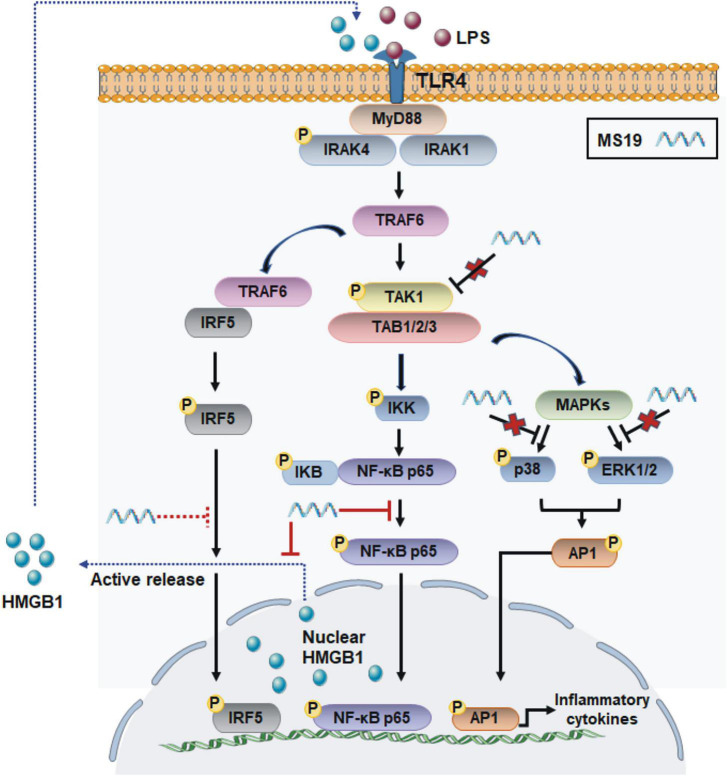
Scheme of the anti-inflammatory mechanisms of MS19 *via* the inhibition of NF-κB signaling and HMGB1 release in LPS/TLR4 pathway. MS19 inhibits the phosphorylation of p65 NF-κB and release of HMGB1 induced by LPS (red solid line); MS19 does not inhibit the phosphorylation of TAK-1, ERK, and p38 MAPK (red cross); our previous study reported that MS19 inhibits the expression and nuclear translocation of IRF5 (red dotted line).

We demonstrated that MS19 remarkably and extensively inhibited multiple PAMPs induced the expression of inflammatory cytokines IL-6 and TNF-α in BMDM and RAW264.7 cells. The inhibition role of TNF-α production of MS19 may be due to block nucleocytoplasmic translocation and secretion of HMGB1, and the inhibition is associated with NF-κB signaling but not the MAPK transduction (Figure 6). Those data may provide a new insight for understanding how MS19 reduces the excessive inflammatory responses in the ALI mouse model induced and excessive cytokine-mediated lethal shock syndrome model. We know that both cytokines TNF-α and HMGB1 were known to be the central mediator of cytokine storm in severe COVID-19 disease (Chen et al., 2020). Kridin et al. (2021) recently analyzed outcomes of COVID-19 in patients with psoriasis treated with anti-TNF biologics and found that TNF blockade significantly decreased the risk of COVID-19-associated hospitalization (Ablamunits and Lepsy, 2022). HMGB1 inhibitors are also promising drug candidates for the treatment of patients suffering from COVID-19. Clinical trials are urgently needed to test different TNF-α/NF-κB inhibitors or HMGB1 antibodies for treatment or prevention of severe COVID-19 cases. Thus, our findings on MS19 in controlling TNF-a production and HMGB1 secretion may provide potential therapeutic application involving targeting cytokines TNF-α- and HMGB1-mediated overactivated inflammation injury induced by SARS-CoV-2.

## Data availability statement

The original contributions presented in the study are included in the article/Supplementary material, further inquiries can be directed to the corresponding authors.

## Ethics statement

The animal study was reviewed and approved by the ethics committee of the College of Basic Medical Sciences of Jilin University.

## Author contributions

CZ and HuiW conducted all the experiments. SS and HRW contributed to the writing of the manuscript. MY and MF provided the ideas and revised the draft manuscript. YS and HuaW approved the version to be published. All authors read and approved the final manuscript.
