# Supplementary material for: A microsatellite DNA-derived oligodeoxynucleotide attenuates lipopolysaccharide-induced acute lung injury in mice by inhibiting the HMGB1-TLR4-NF-κB signaling pathway
Source: Front Microbiol. 2022 Aug 4;13:964112. doi: 10.3389/fmicb.2022.964112 (PMC9386506; doi:10.3389/fmicb.2022.964112)
Supplement: Supplementary file 1 [file Data_Sheet_1.docx]

Methods:

Total RNA isolation and qRT-PCR in RAW 264.7 cells

The RAW 264.7 cell pellets were used for RNA extraction. Total RNA was extracted from cells using TRIzol reagent (Solarbio, Beijing, China) and reverse-transcribed into cDNA using the Prime Script RT Reagent Kit with gDNA Eraser (Takara Bio, Dalian, China). TLR4, IRF3, MyD88 and TARF6 mRNA levels were evaluated using qRT-PCR on a LightCycler 480 Realtime Detection System (Roche, Basel, Switzerland). Reaction mixtures consisted of 10 μL of 2 × SYBR Green II PCR Mix (Takara Bio), 25 μmol/L forward and reverse primers, and 2 μL of template; double-distilled H_2_O was added to achieve a final volume of 20 μL. The PCR reaction conditions were as follows: 95 ℃ for 30 s, followed by 40 cycles of 95 ℃ for 10 s, 60 ℃ for 20 s, and 72 ◦C for 10 s. β-actin was used as an internal reference gene. Expression levels of target genes were calculated as relative values using the 2^-ΔΔCt^ method. PCR primers (Table S1) were obtained from Shanghai Genechem Co., Ltd. (Shanghai, China).

Table S1. Primers used for qRT-PCR analysis

| Primer name | Primer sequences (5’-3’) |
| --- | --- |
| mTLR4-F | AGCTTCTCCAATTTTTCAGAACTTC |
| mTLR4-R | TGAGAGGTGGTGTAAGCCATGC |
| mIRF3-F | CGGAAAGAAGTGTTGCGGTTAGC |
| mIRF3-R | CAGGCTGCTTTTGCCATTGGTG |
| mMyD88-F | ACCTGTGTCTGGTCCATTGCCA |
| mMyD88-R | GCTGAGTGCAAACTTGGTCTGG |
| mTARF6-F | TTTCCCTGACGGTAAAGTGCCC |
| mTARF6-R | ACCTGGCACTTCTGGAAAGGAC |
| mβ-actin-F | TGGTTACAGGAAGTCCCTCAC |
| mβ-actin-R | ACAGAAGCAATGCTGTCACCTT |

Figure S1


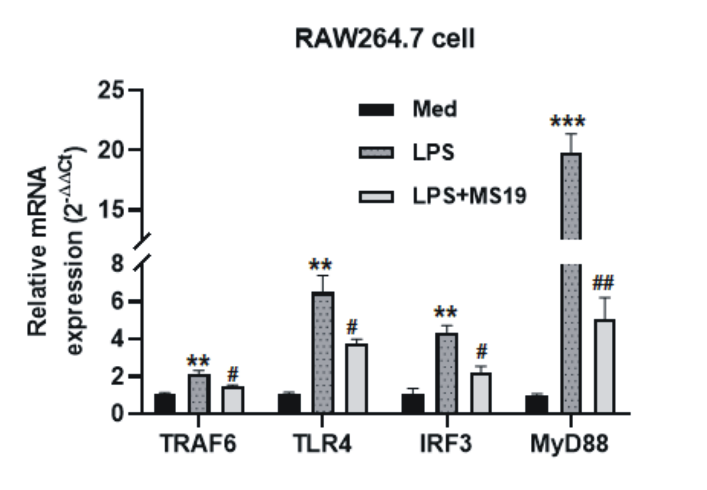


Figure S1. Effect of MS19 on the mRNA expression of important molecules in MyD88 signaling pathways with LPS stimulation in RAW264.7 cells. RAW264.7 cells were treated with LPS in the presence or absence of MS19 about 30 mins, then the cells were collected to evaluate the expression of TARF6, TLR4, IRF3 and MyD88 mRNAs with qRT-PCR. The values are presented as the means ± SEM of three independent experiments. *p < 0.05, **p < 0.01, and ***p < 0.001, versus medium group; ^#^p < 0.05, ^##^p < 0.01, versus LPS group.
