# Supplementary material for: A microsatellite DNA-derived oligodeoxynucleotide attenuates lipopolysaccharide-induced acute lung injury in mice by inhibiting the HMGB1-TLR4-NF-κB signaling pathway
Source: Front Microbiol. 2022 Aug 4;13:964112. doi: 10.3389/fmicb.2022.964112 (PMC9386506; doi:10.3389/fmicb.2022.964112)
Supplement: Supplementary file 2 [file Data_Sheet_2.zip › Raw data/Figure 5 raw data/lung inflammatory pictures.PPTX]

## Slide 1
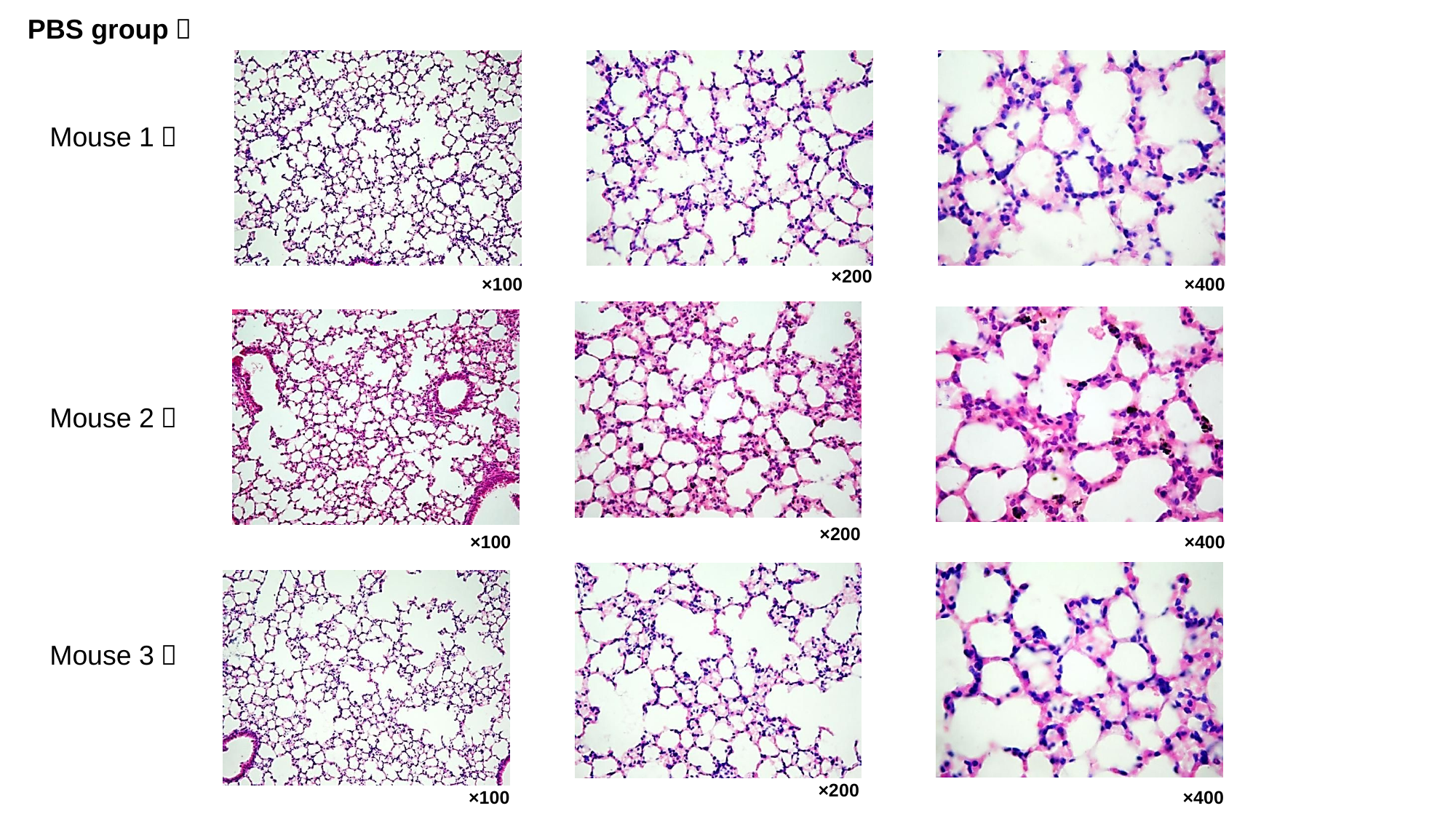

PBS group：
Mouse 1：
×200
×400
×100
Mouse 2：
×200
×400
×100
Mouse 3：
×200
×400
×100

## Slide 2
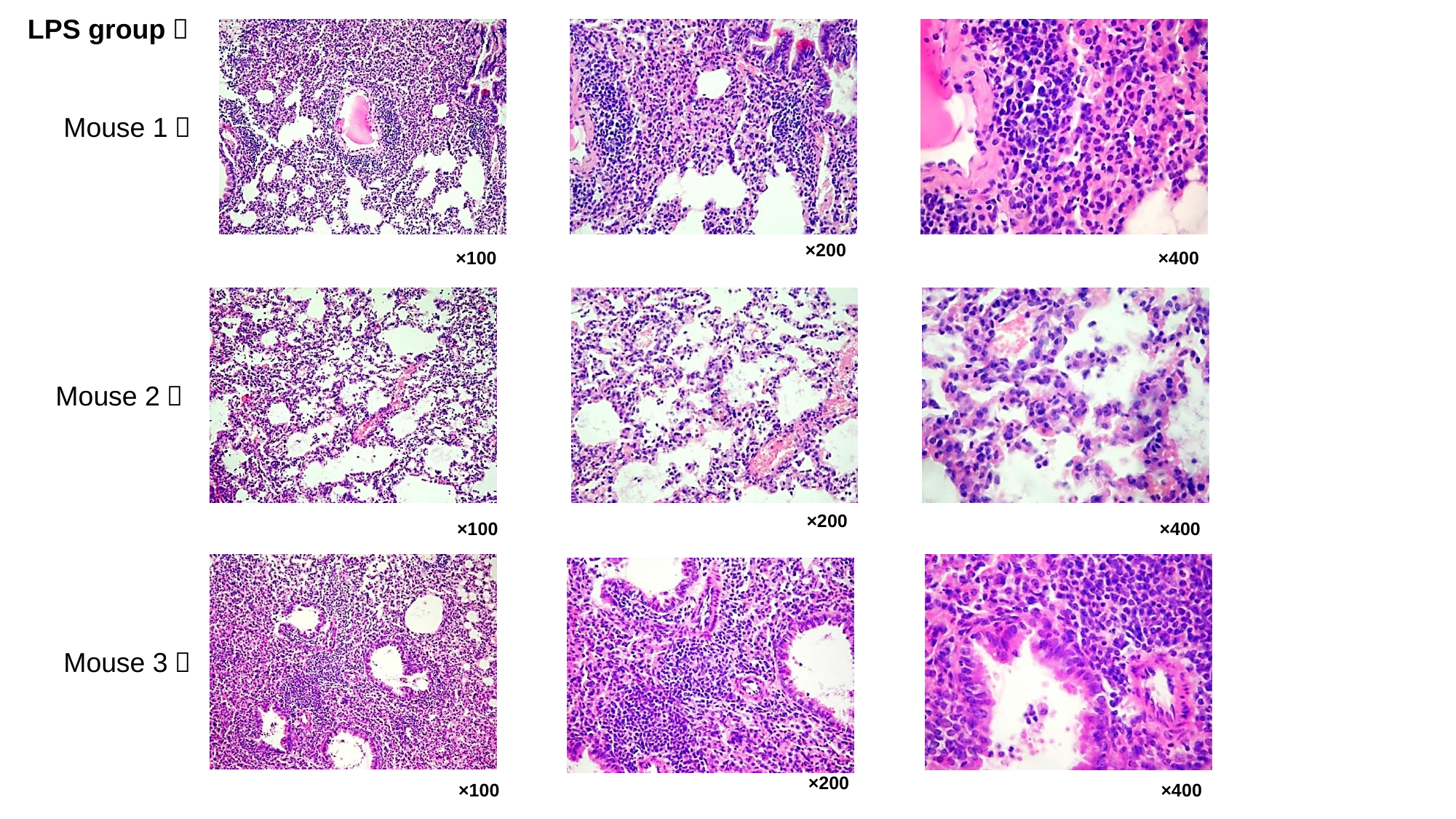

LPS group：
Mouse 1：
×200
×400
×100
Mouse 2：
×200
×400
×100
Mouse 3：
×200
×400
×100

## Slide 3
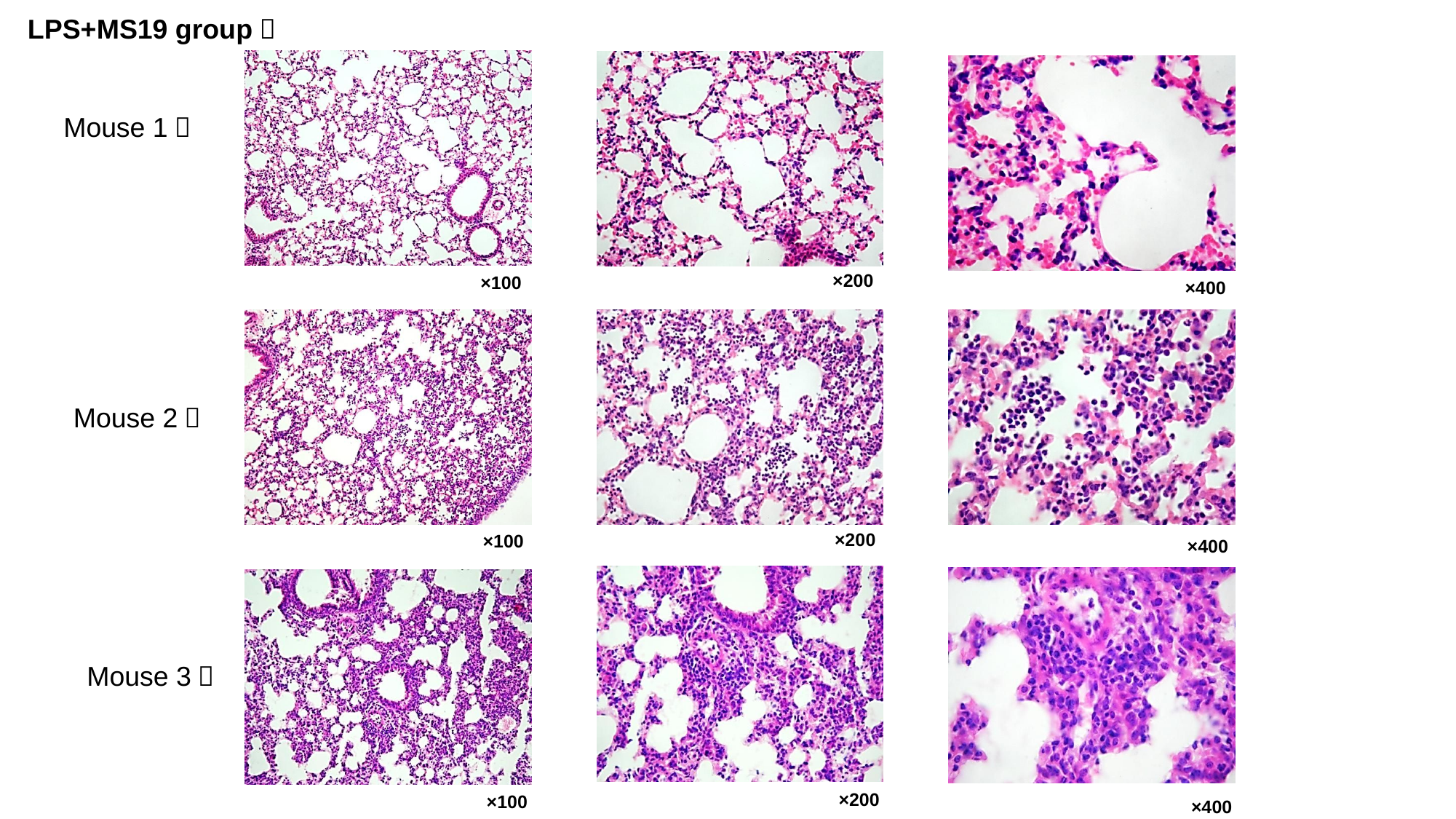

LPS+MS19 group：
Mouse 1：
×200
×100
×400
Mouse 2：
×200
×100
×400
Mouse 3：
×200
×100
×400
