# Supplementary figures and images for: A microsatellite DNA-derived oligodeoxynucleotide attenuates lipopolysaccharide-induced acute lung injury in mice by inhibiting the HMGB1-TLR4-NF-κB signaling pathway
Source: Front Microbiol. 2022 Aug 4;13:964112. doi: 10.3389/fmicb.2022.964112 (PMC9386506; doi:10.3389/fmicb.2022.964112)

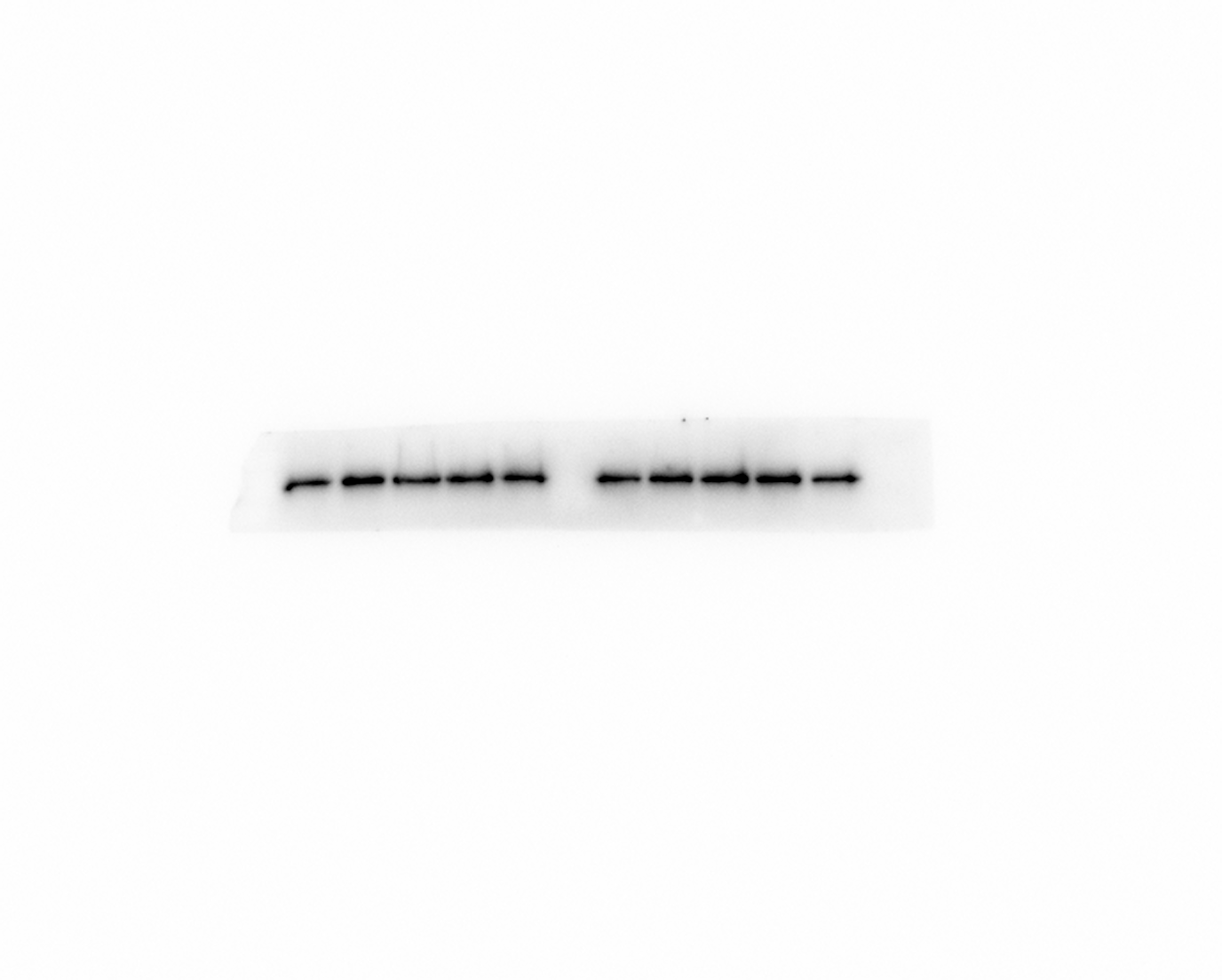

Supplement: Supplementary file 2 [file Data_Sheet_2.zip › Raw data/Figure 2 raw data/PR8-P65/20191227_1034_03 P65 (lane 1-5).Tif]

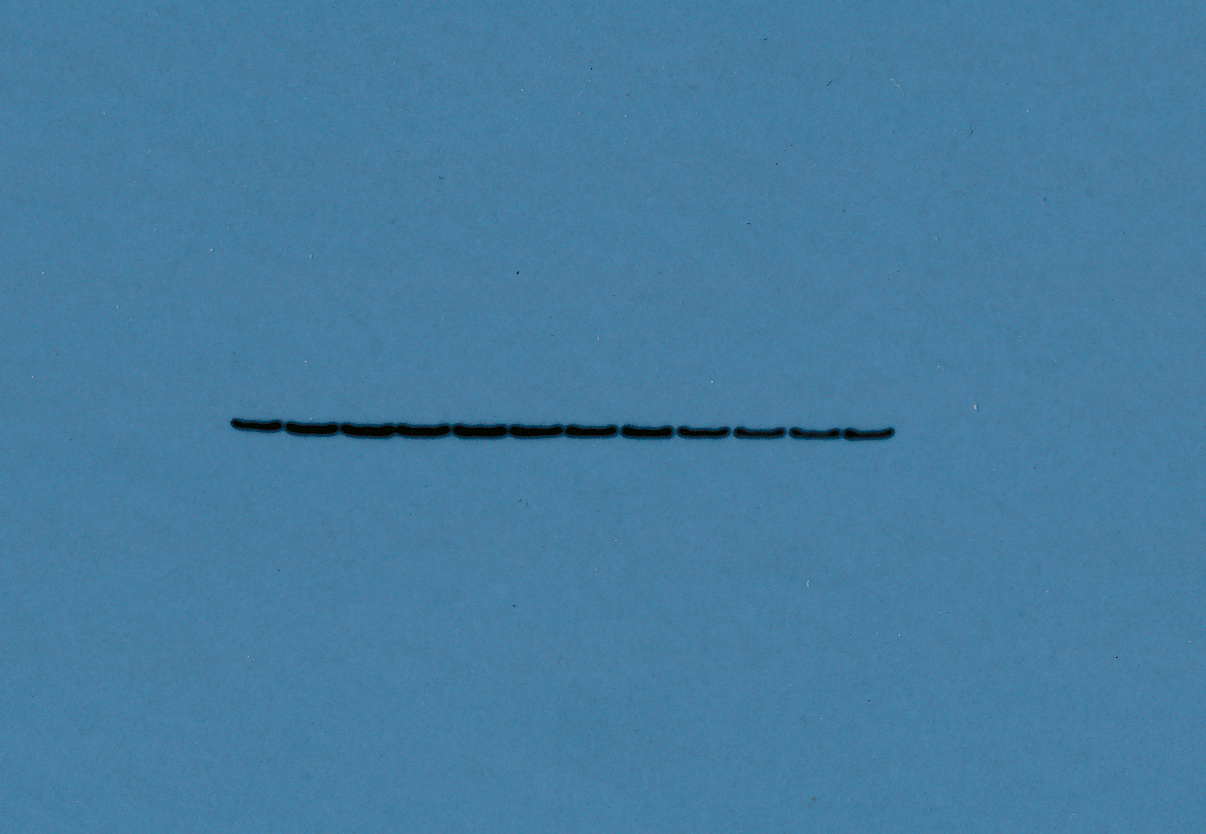

Supplement: Supplementary file 2 [file Data_Sheet_2.zip › Raw data/Figure 3 raw data/b-actin (lane 1-6).tif]

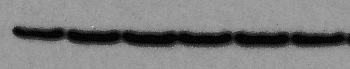

Supplement: Supplementary file 2 [file Data_Sheet_2.zip › Raw data/Figure 3 raw data/b-actin-c.tif]

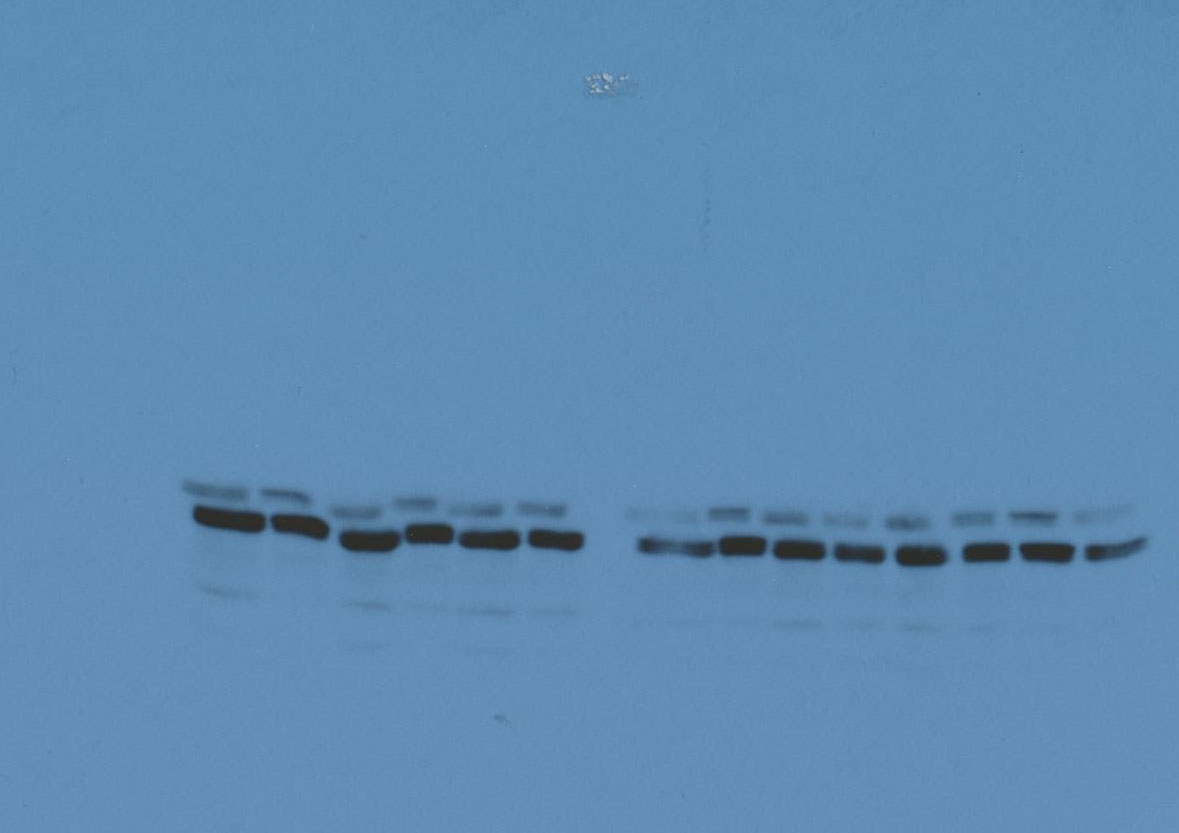

Supplement: Supplementary file 2 [file Data_Sheet_2.zip › Raw data/Figure 3 raw data/erk total (lane 1-6).jpg]

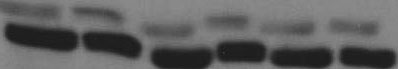

Supplement: Supplementary file 2 [file Data_Sheet_2.zip › Raw data/Figure 3 raw data/erk total-c.jpg]

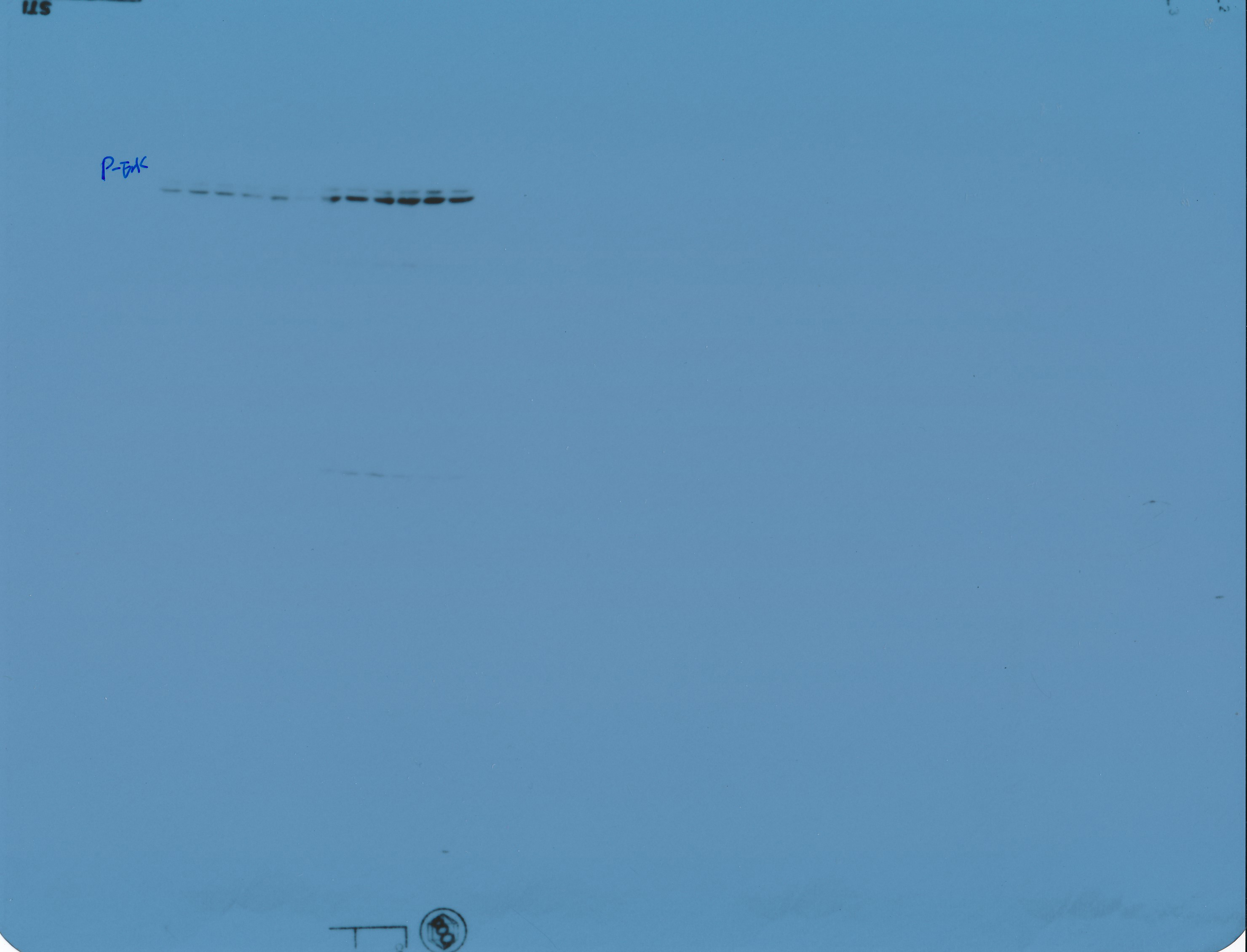

Supplement: Supplementary file 2 [file Data_Sheet_2.zip › Raw data/Figure 3 raw data/p-erk (lane 1-6).jpg]

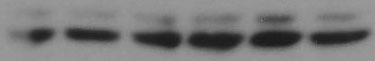

Supplement: Supplementary file 2 [file Data_Sheet_2.zip › Raw data/Figure 3 raw data/p-erk-c.jpg]

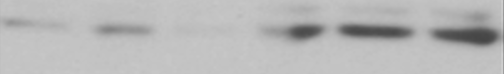

Supplement: Supplementary file 2 [file Data_Sheet_2.zip › Raw data/Figure 3 raw data/p-erk.tif]

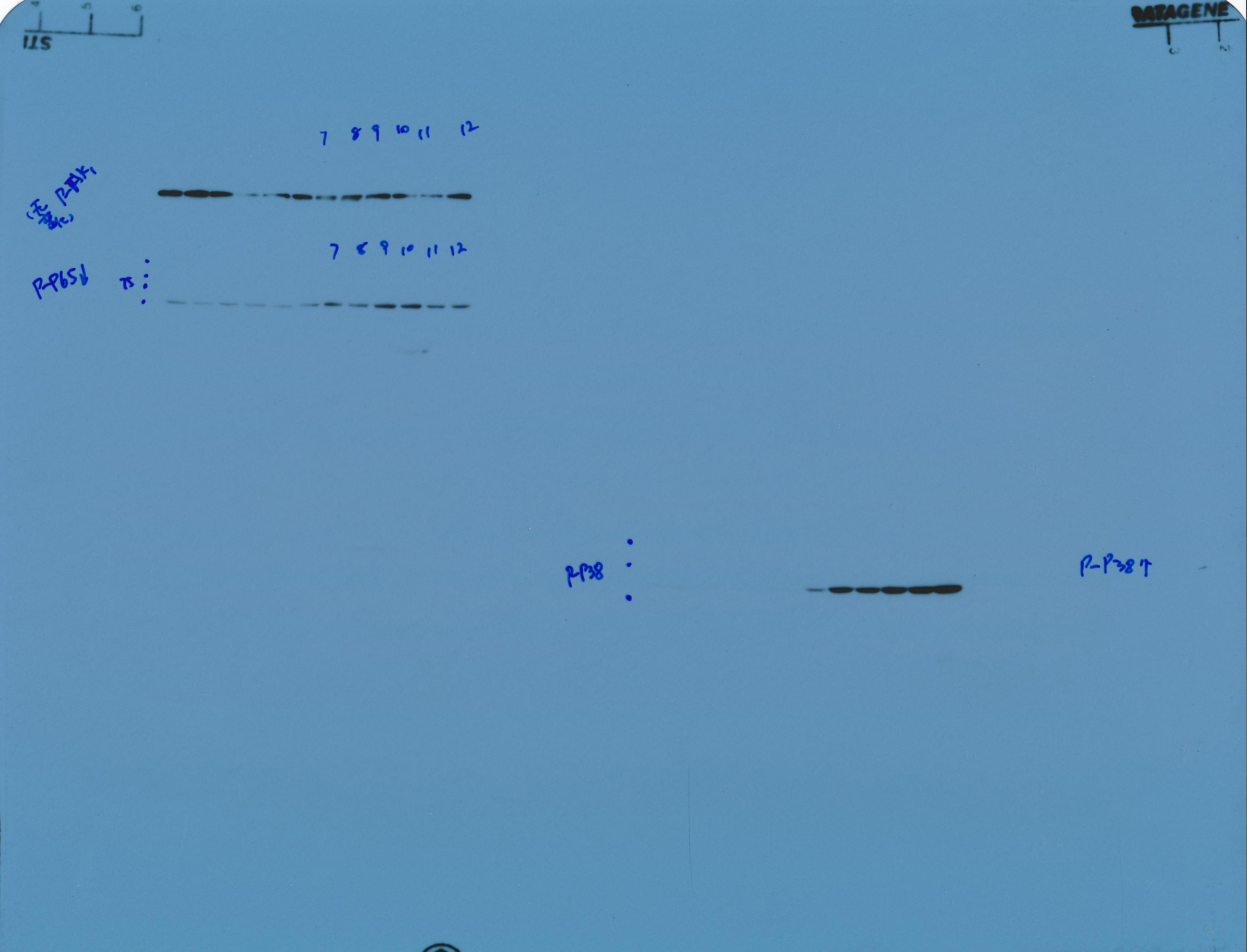

Supplement: Supplementary file 2 [file Data_Sheet_2.zip › Raw data/Figure 3 raw data/p-p38.jpg]

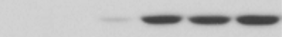

Supplement: Supplementary file 2 [file Data_Sheet_2.zip › Raw data/Figure 3 raw data/p-p38.tif]

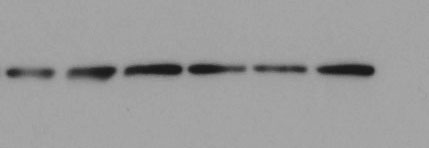

Supplement: Supplementary file 2 [file Data_Sheet_2.zip › Raw data/Figure 3 raw data/p-tak1-1.jpg]

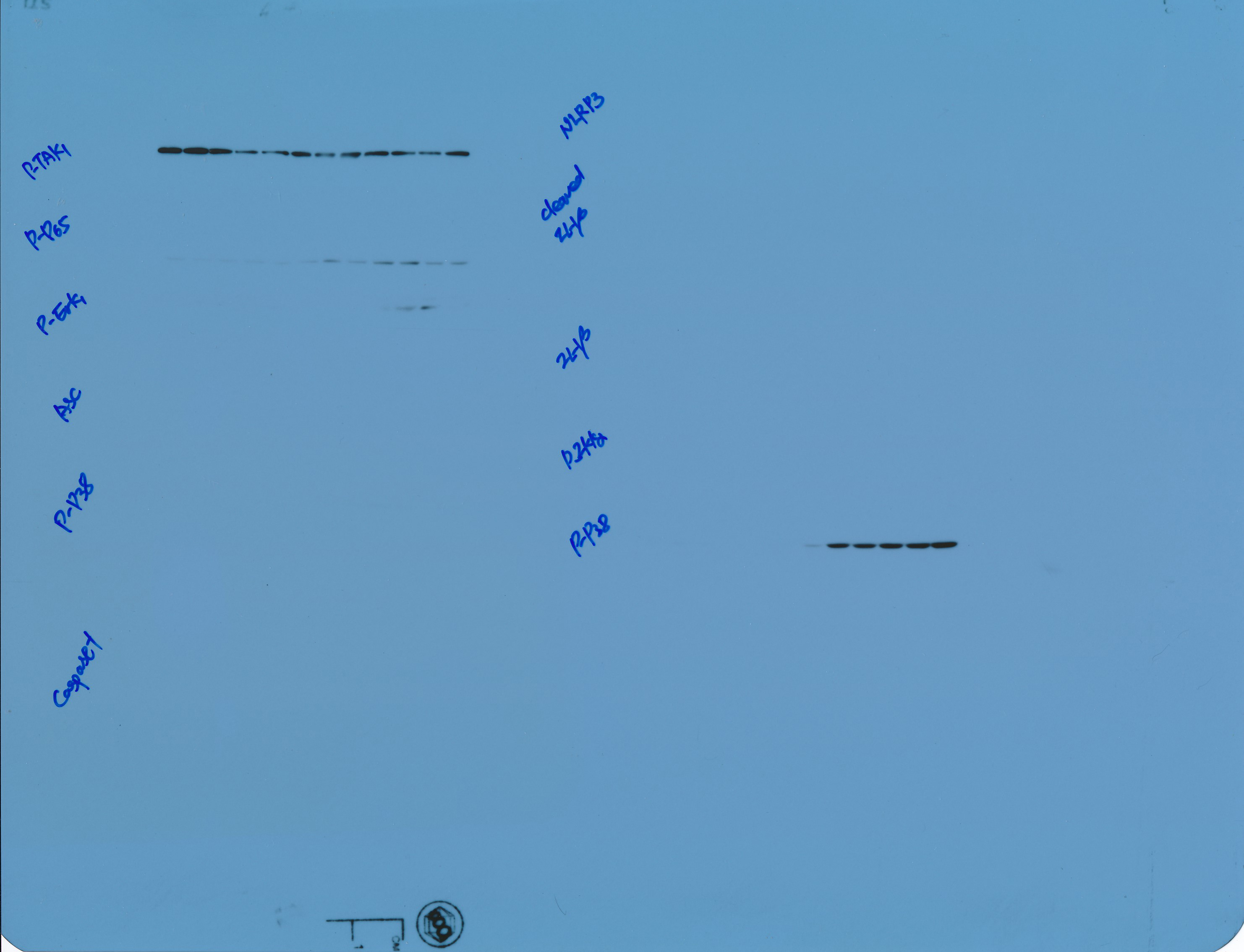

Supplement: Supplementary file 2 [file Data_Sheet_2.zip › Raw data/Figure 3 raw data/p-tak1.jpg]

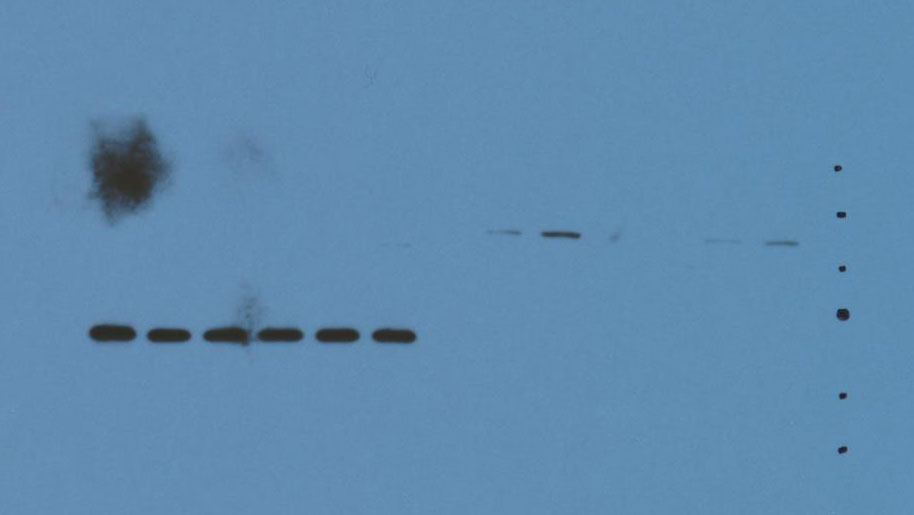

Supplement: Supplementary file 2 [file Data_Sheet_2.zip › Raw data/Figure 3 raw data/p38 (lane 1-6).jpg]

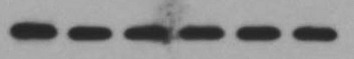

Supplement: Supplementary file 2 [file Data_Sheet_2.zip › Raw data/Figure 3 raw data/p38-c.tif]

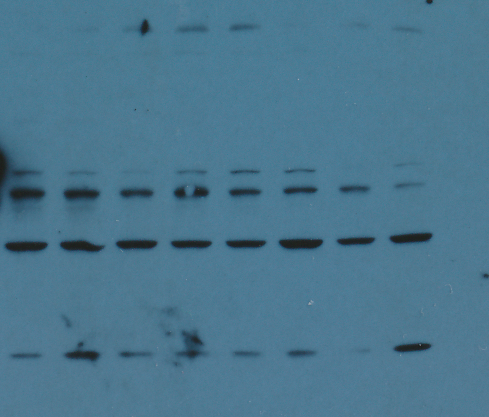

Supplement: Supplementary file 2 [file Data_Sheet_2.zip › Raw data/Figure 3 raw data/tak-total (lane 1-6).tif]

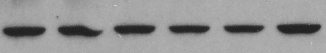

Supplement: Supplementary file 2 [file Data_Sheet_2.zip › Raw data/Figure 3 raw data/tak.tif]

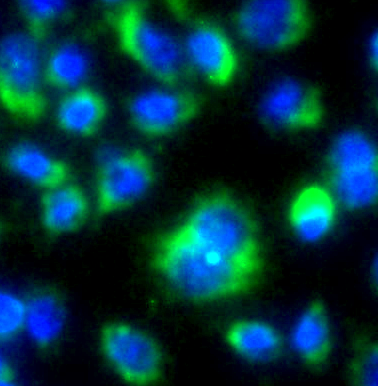

Supplement: Supplementary file 2 [file Data_Sheet_2.zip › Raw data/Figure 4 raw data/LPS group/10.LPS composite.tif]

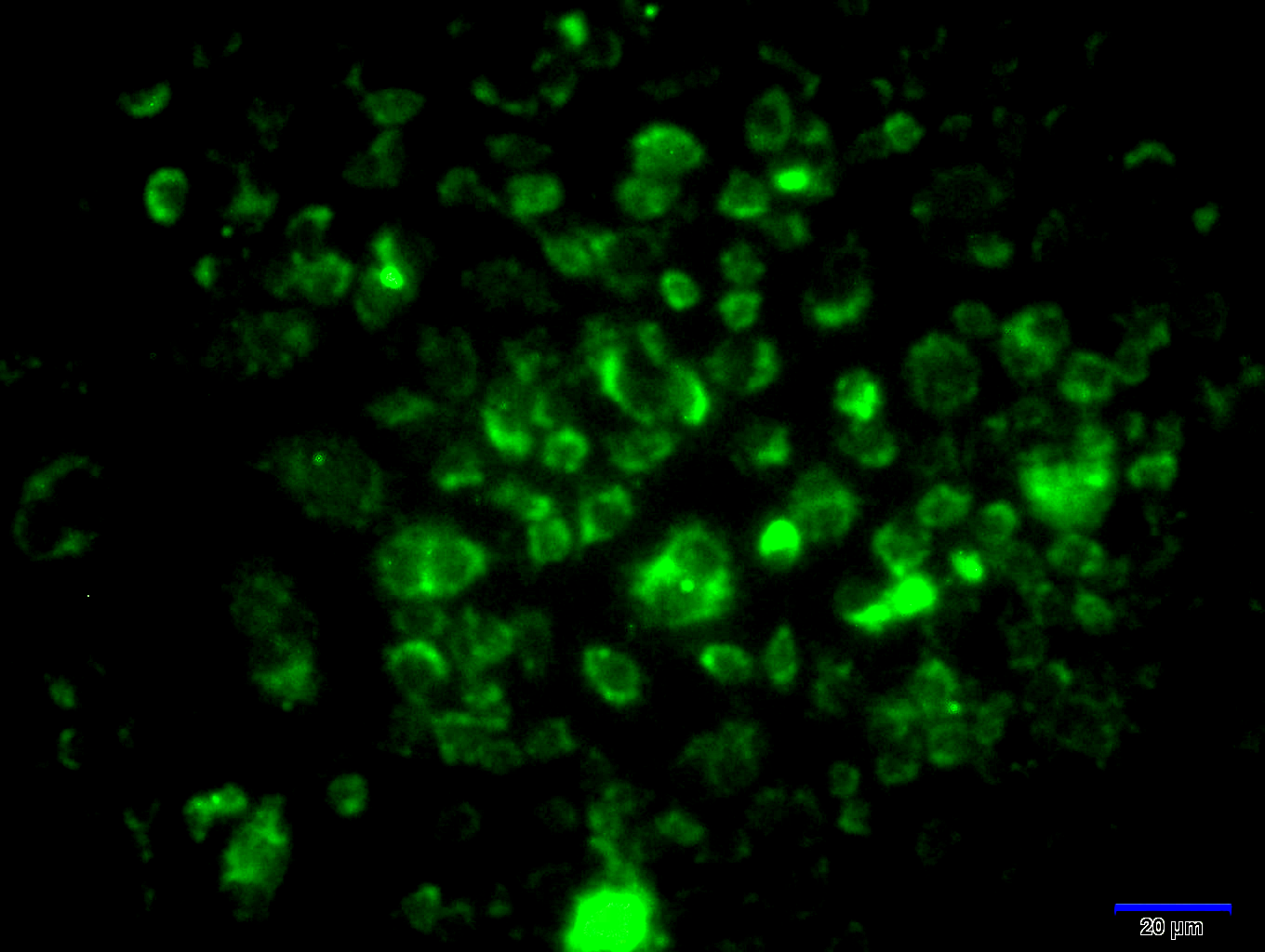

Supplement: Supplementary file 2 [file Data_Sheet_2.zip › Raw data/Figure 4 raw data/LPS group/6. LPS-G.tif]

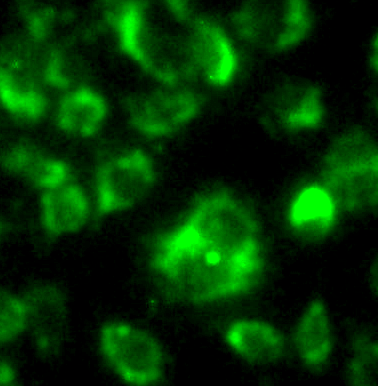

Supplement: Supplementary file 2 [file Data_Sheet_2.zip › Raw data/Figure 4 raw data/LPS group/7. LPS-G-c.tif]

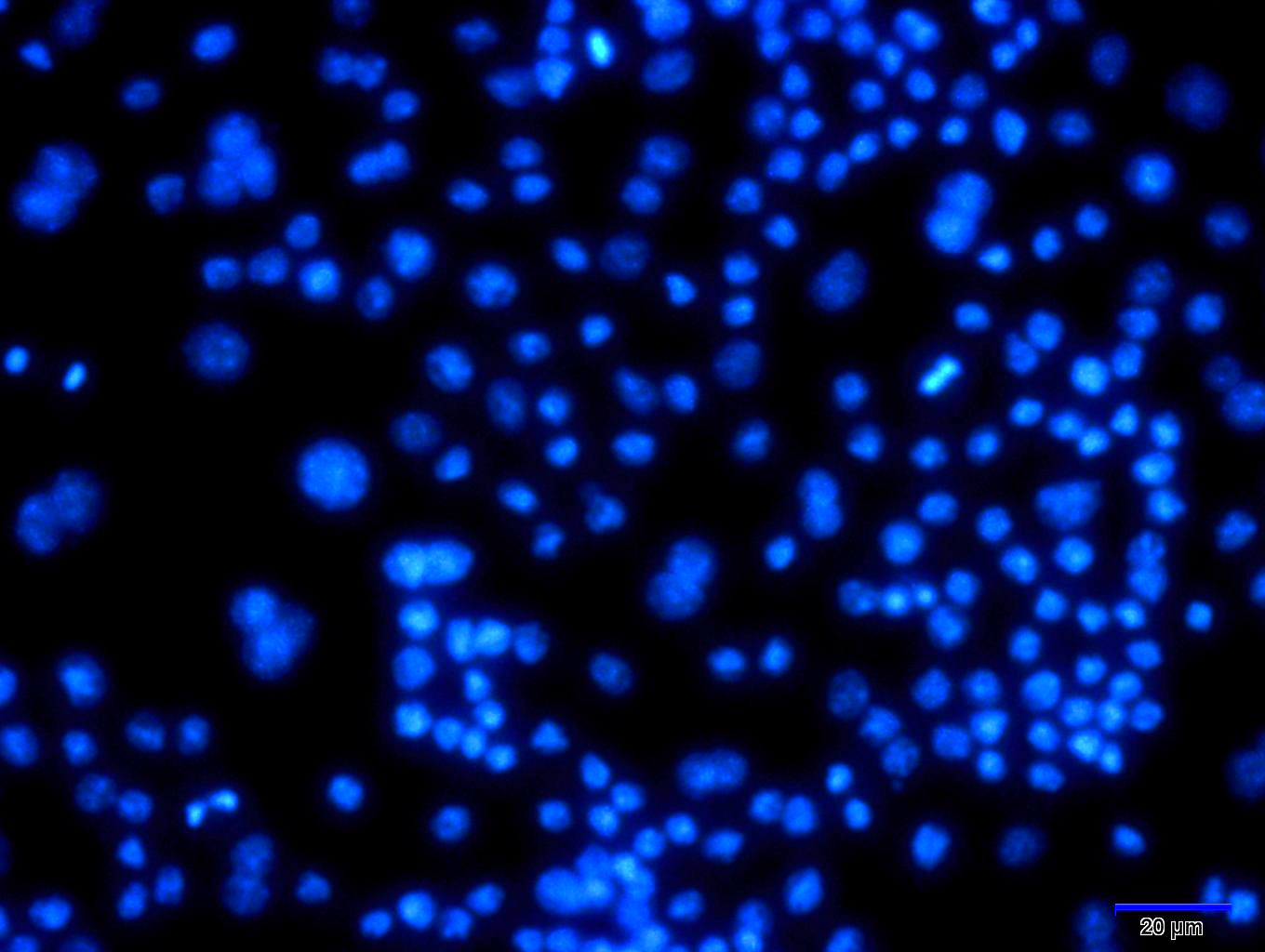

Supplement: Supplementary file 2 [file Data_Sheet_2.zip › Raw data/Figure 4 raw data/LPS group/8. LPS-L.tif]

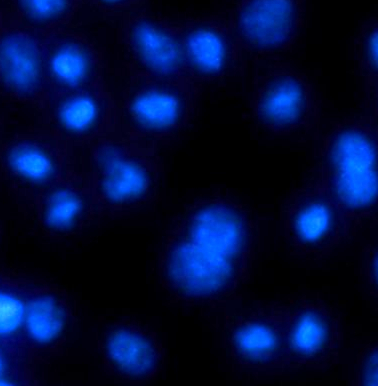

Supplement: Supplementary file 2 [file Data_Sheet_2.zip › Raw data/Figure 4 raw data/LPS group/9. LPS-1cc.tif]

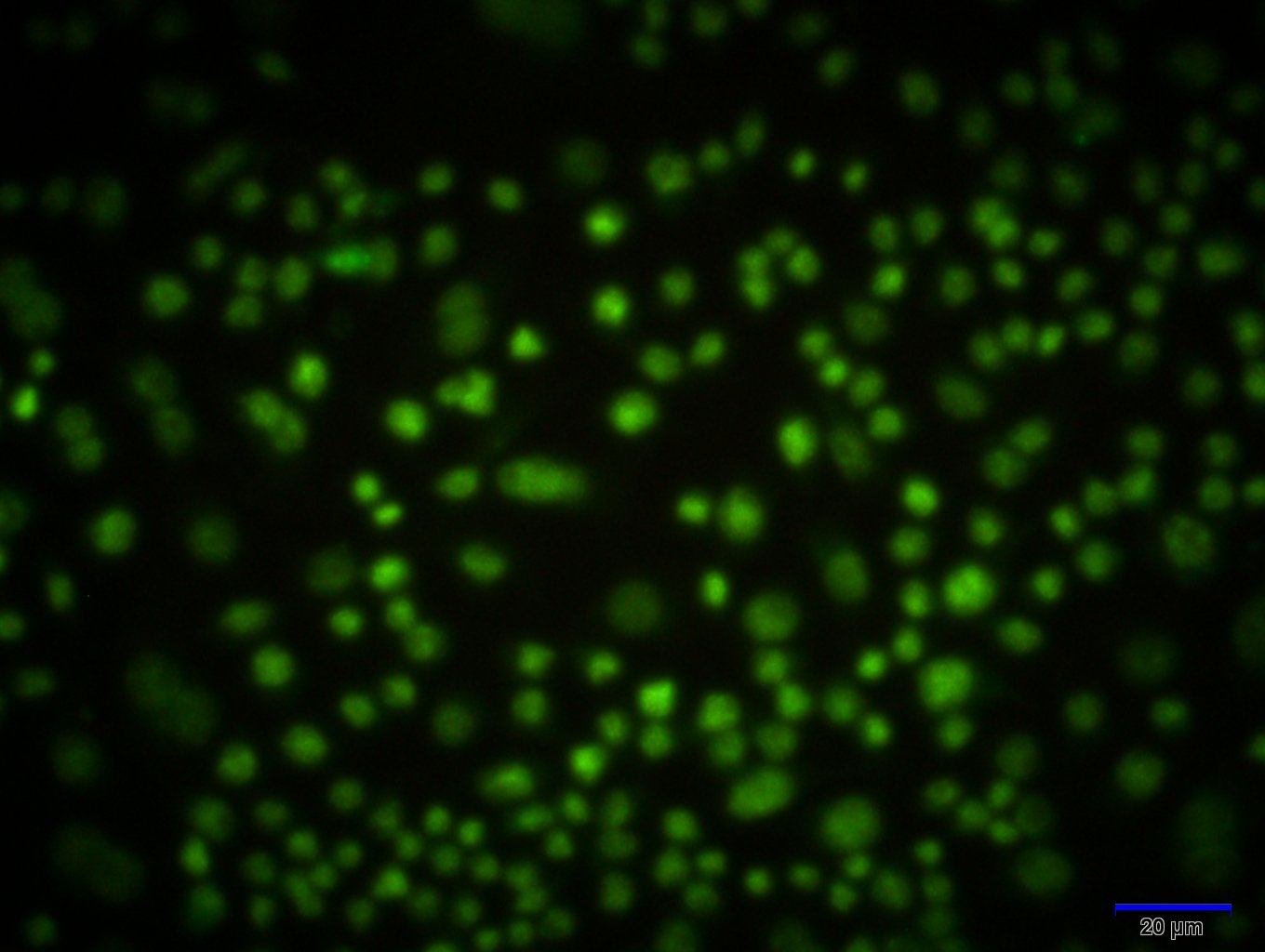

Supplement: Supplementary file 2 [file Data_Sheet_2.zip › Raw data/Figure 4 raw data/LPS+MS19 group/11. LPSMS19-G.tif]

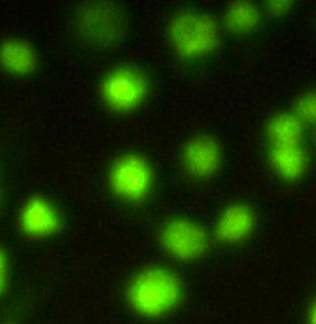

Supplement: Supplementary file 2 [file Data_Sheet_2.zip › Raw data/Figure 4 raw data/LPS+MS19 group/12. LPSMS19-G-c.tif]

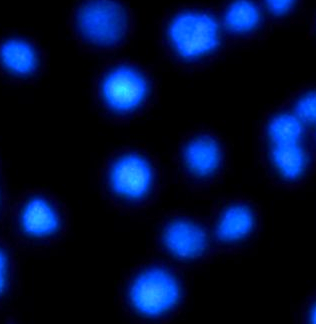

Supplement: Supplementary file 2 [file Data_Sheet_2.zip › Raw data/Figure 4 raw data/LPS+MS19 group/13. LPSMS19-L-1c.tif]

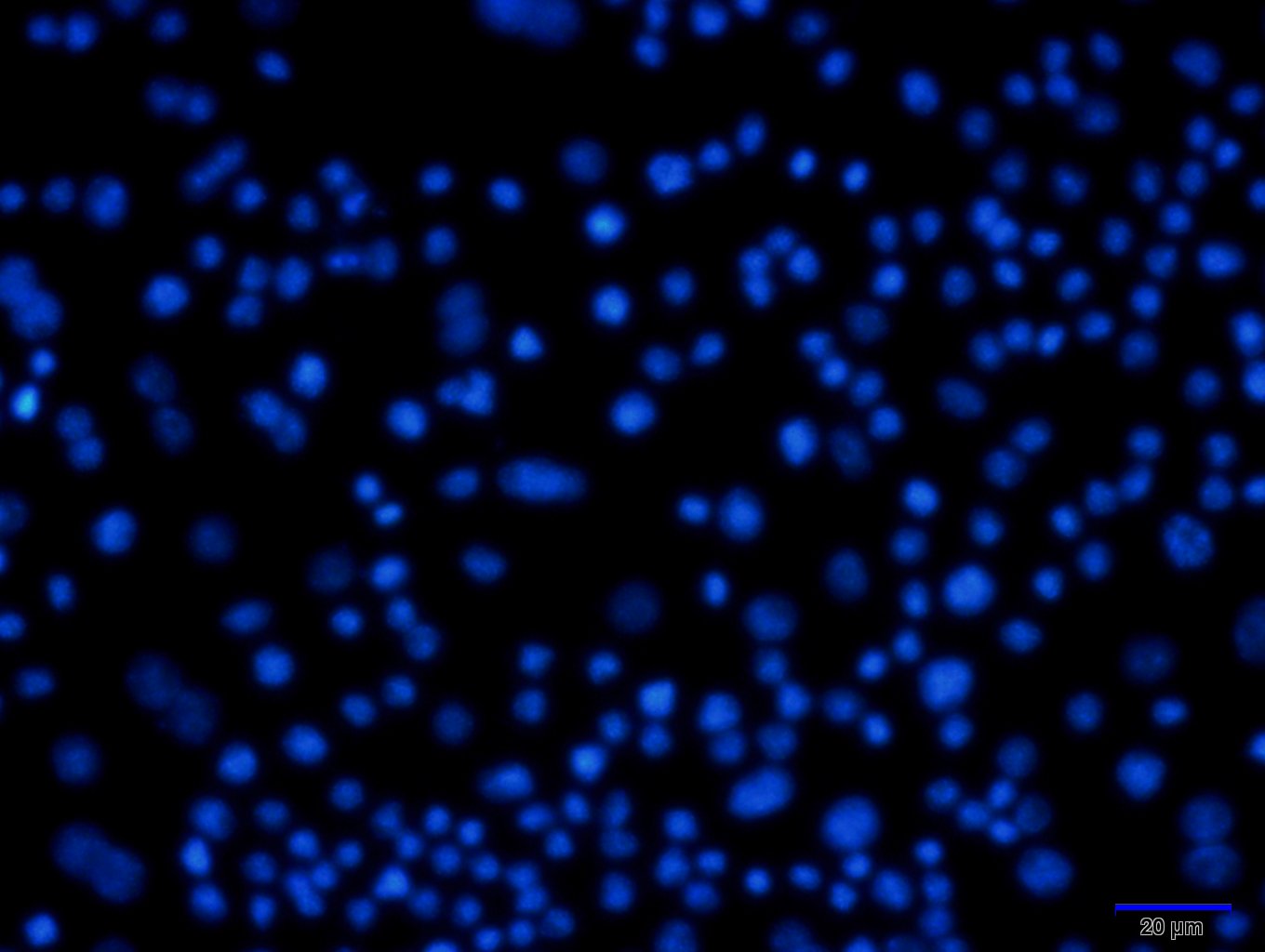

Supplement: Supplementary file 2 [file Data_Sheet_2.zip › Raw data/Figure 4 raw data/LPS+MS19 group/14. LPSMS19-L.tif]

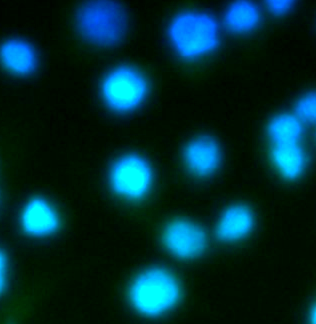

Supplement: Supplementary file 2 [file Data_Sheet_2.zip › Raw data/Figure 4 raw data/LPS+MS19 group/15. LPS+MS19 Composite.tif]

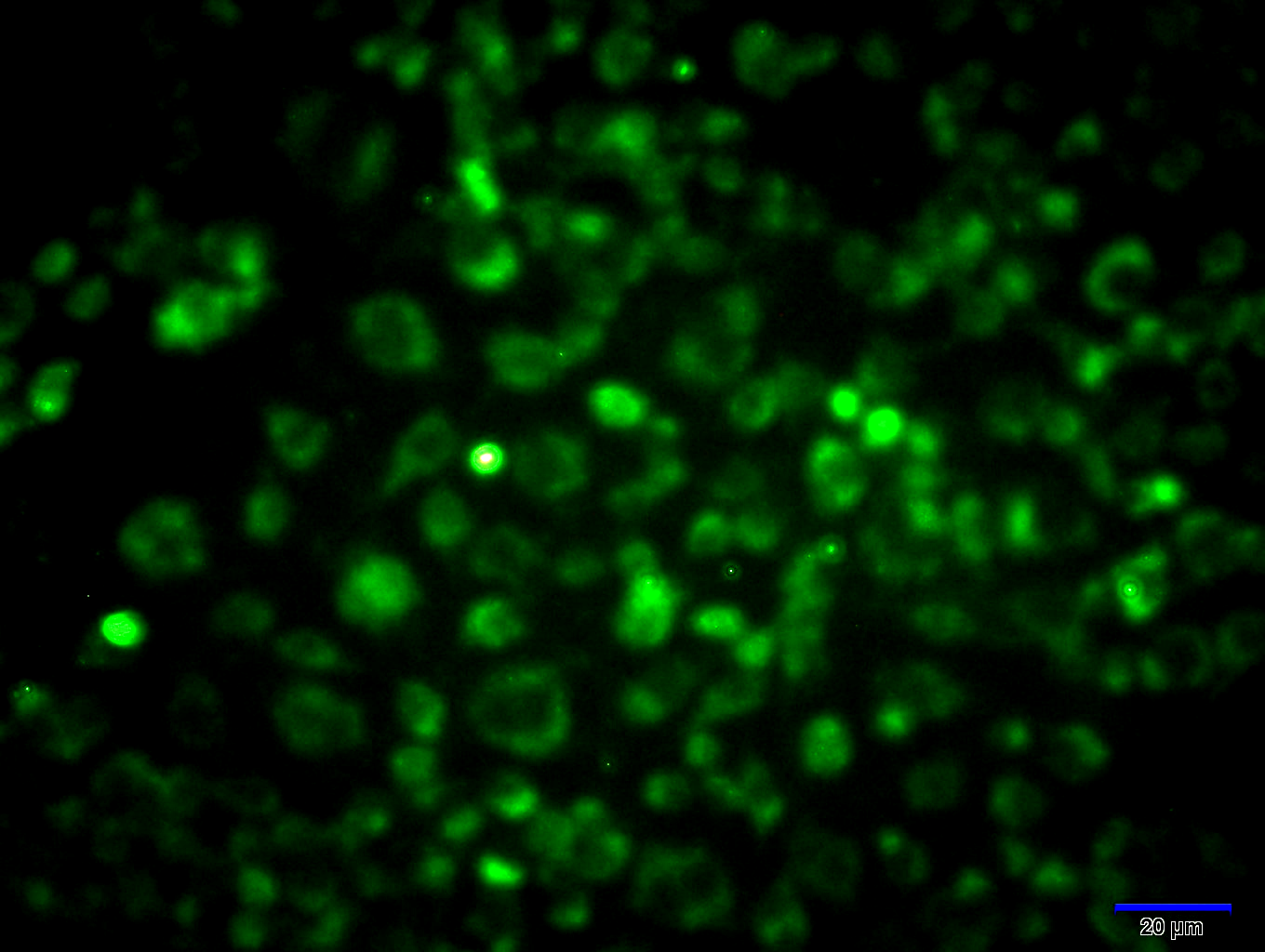

Supplement: Supplementary file 2 [file Data_Sheet_2.zip › Raw data/Figure 4 raw data/LPS+MS19-C group/16 LPS+MS19-C-1.tif]

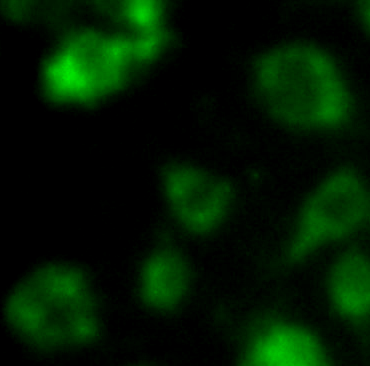

Supplement: Supplementary file 2 [file Data_Sheet_2.zip › Raw data/Figure 4 raw data/LPS+MS19-C group/17 LPS-+MS19-C-1c.tif]

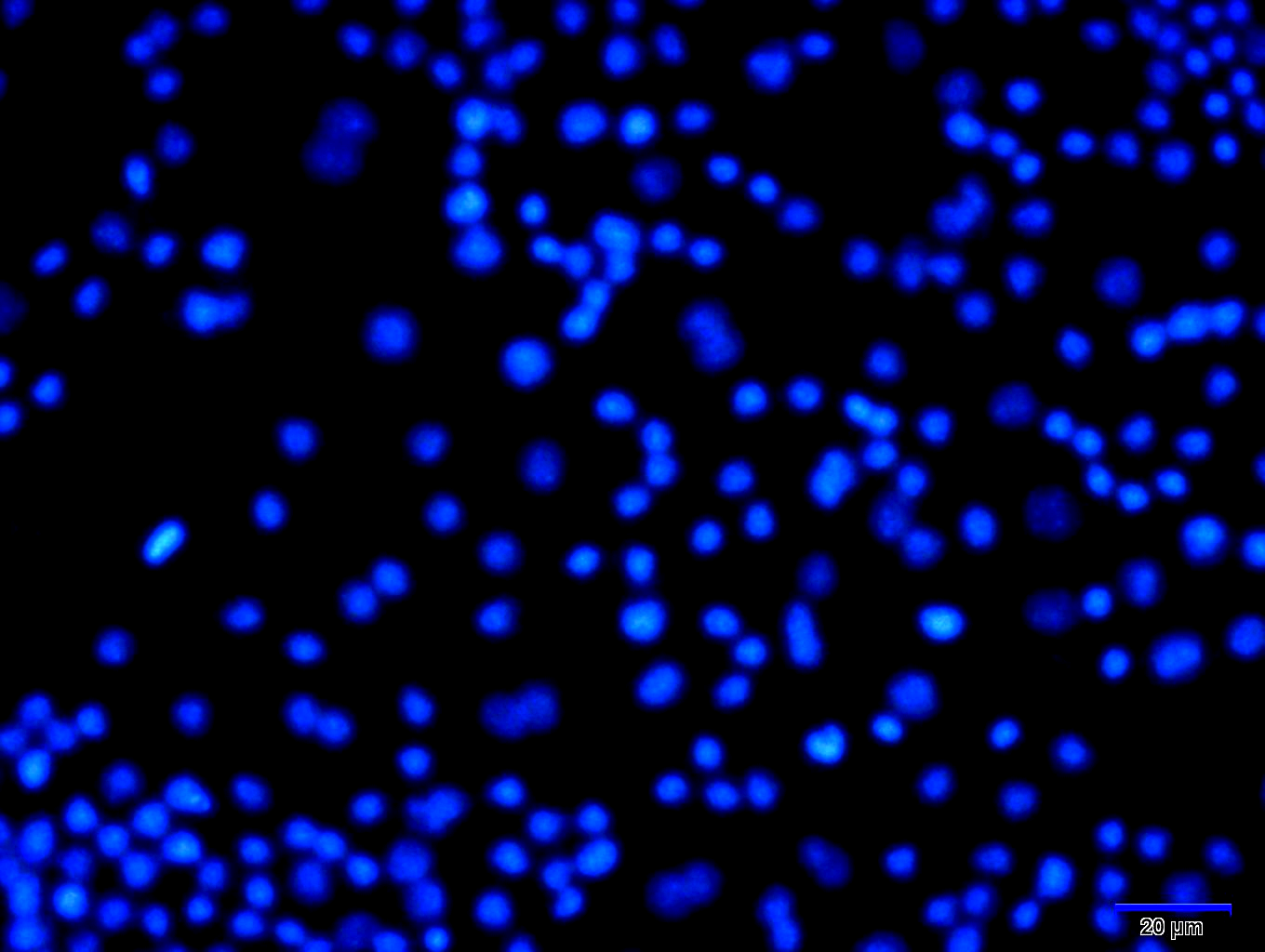

Supplement: Supplementary file 2 [file Data_Sheet_2.zip › Raw data/Figure 4 raw data/LPS+MS19-C group/18 LPS+MS19-C-1.tif]

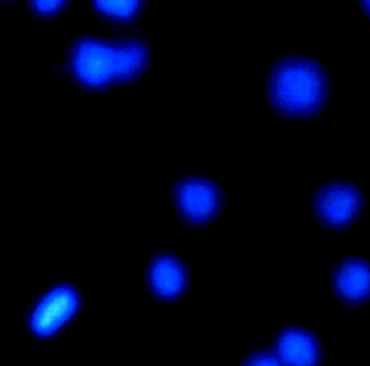

Supplement: Supplementary file 2 [file Data_Sheet_2.zip › Raw data/Figure 4 raw data/LPS+MS19-C group/19.LPS+MS19-C-L-1c.tif]

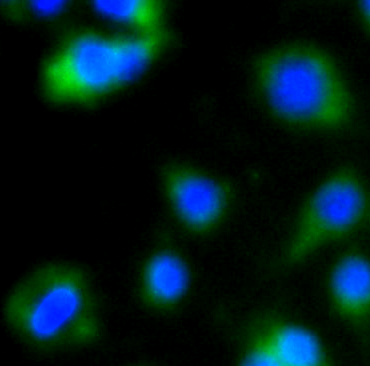

Supplement: Supplementary file 2 [file Data_Sheet_2.zip › Raw data/Figure 4 raw data/LPS+MS19-C group/20 LPS+MS19C Composite.tif]

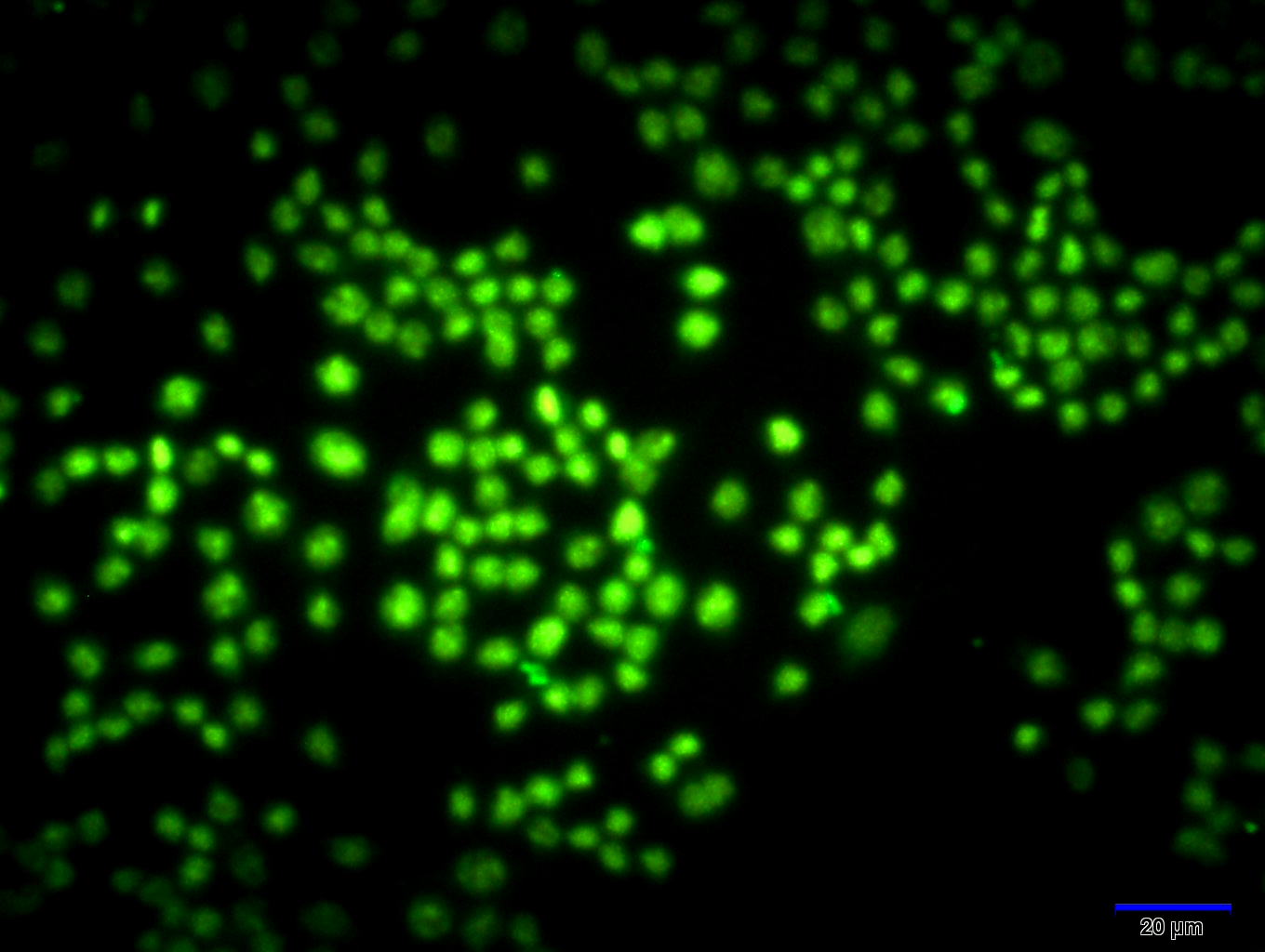

Supplement: Supplementary file 2 [file Data_Sheet_2.zip › Raw data/Figure 4 raw data/medium group/1. medium-g-1.tif]

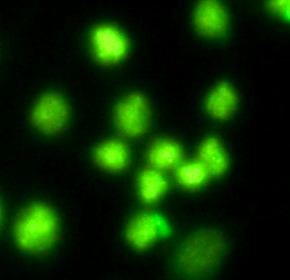

Supplement: Supplementary file 2 [file Data_Sheet_2.zip › Raw data/Figure 4 raw data/medium group/2. medium-g-1c.tif]

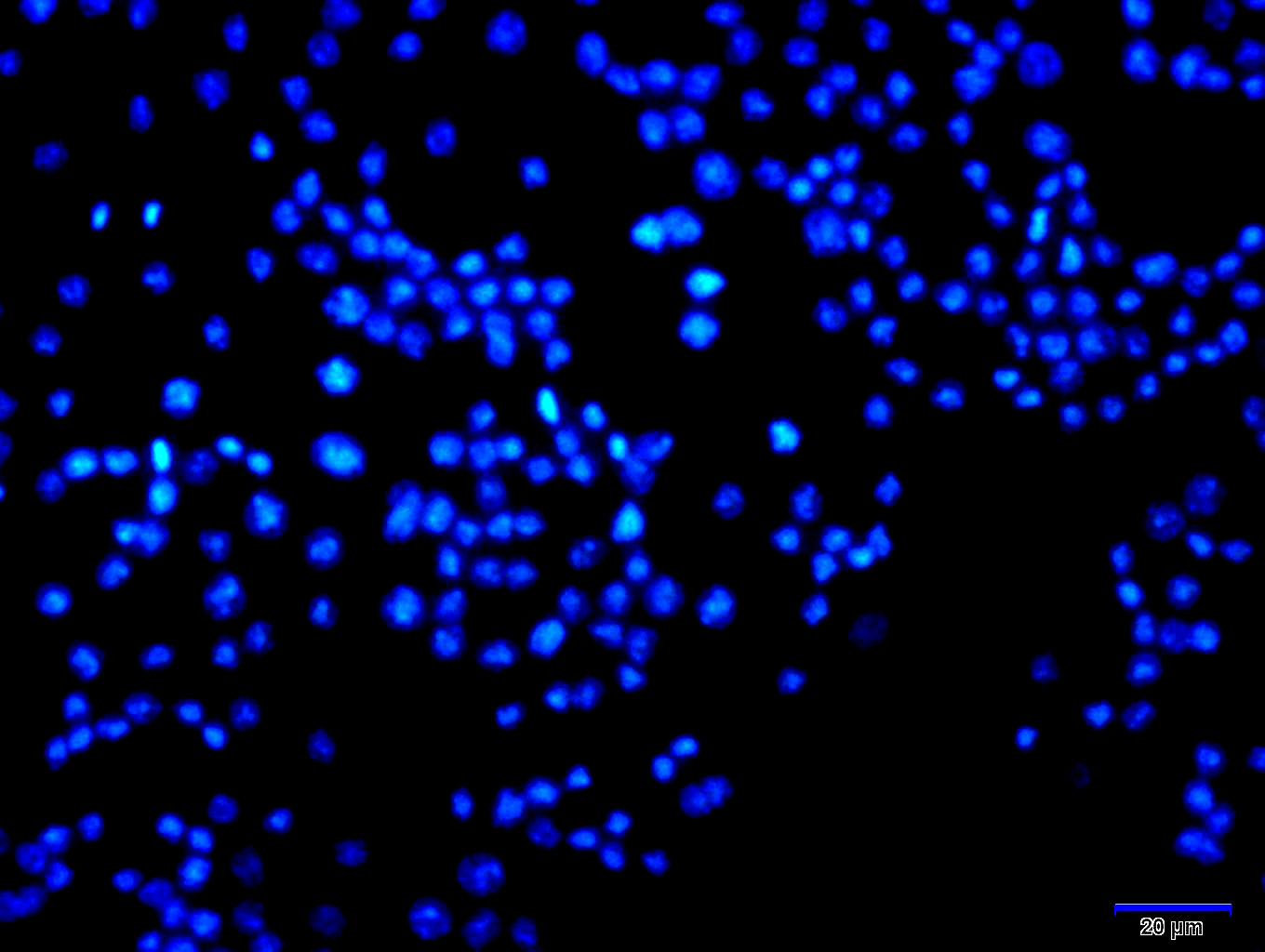

Supplement: Supplementary file 2 [file Data_Sheet_2.zip › Raw data/Figure 4 raw data/medium group/3. medium-l-1.tif]

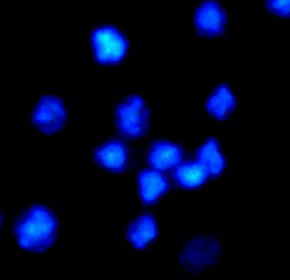

Supplement: Supplementary file 2 [file Data_Sheet_2.zip › Raw data/Figure 4 raw data/medium group/4. medium-l-1c.tif]

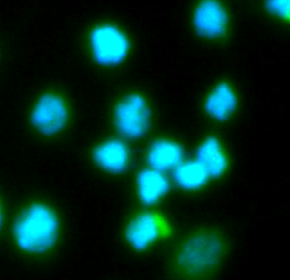

Supplement: Supplementary file 2 [file Data_Sheet_2.zip › Raw data/Figure 4 raw data/medium group/5. medium Composite.tif]
